# Consumption of the Total Western Diet Promotes Colitis and Inflammation-Associated Colorectal Cancer in Mice

**DOI:** 10.3390/nu12020544

**Published:** 2020-02-20

**Authors:** Abby D. Benninghoff, Korry J. Hintze, Stephany P. Monsanto, Daphne M. Rodriguez, Ashli H. Hunter, Sumira Phatak, James J. Pestka, Arnaud J. Van Wettere, Robert E. Ward

**Affiliations:** 1 Department of Animal, Dairy and Veterinary Sciences, 4815 Old Main Hill, Utah State University, Logan, UT 84322, USA; 2 USTAR Applied Nutrition Research, 9815 Old Main Hill, Utah State University, Logan, UT 84322, USA; 3 Department of Nutrition, Dietetics and Food Sciences, 8700 Old Main Hill, Utah State University, Logan, UT 84322, USA; 4 Department of Food Science and Human Nutrition, the Institute for Integrative Toxicology, and the Department of Microbiology and Molecular Genetics, Michigan State University, East Lansing, MI 48824, USA

**Keywords:** Western diet, colitis, inflammation, colorectal cancer, calcium, vitamin D, transcript profiling

## Abstract

Consumption of a Western type diet is a known risk factor for colorectal cancer. Our group previously developed the total Western diet (TWD) for rodents with energy and nutrient profiles that emulate a typical Western diet. In this study, we tested the hypothesis that consumption of the TWD would enhance colitis, delay recovery from gut injury and promote colon tumorigenesis. In multiple experiments using the azoxymethane + dextran sodium sulfate or *Apc^Min/+^* mouse models of colitis-associated colorectal carcinogenesis (CAC), we determined that mice fed TWD experienced more severe and more prolonged colitis compared to their counterparts fed the standard AIN93G diet, ultimately leading to markedly enhanced colon tumorigenesis. Additionally, this increased tumor response was attributed to the micronutrient fraction of the TWD, and restoration of calcium and vitamin D to standard amounts ameliorated the tumor-promoting effects of TWD. Finally, exposure to the TWD elicited large scale, dynamic changes in mRNA signatures of colon mucosa associated with interferon (IFN) response, inflammation, innate immunity, adaptive immunity, and antigen processing pathways, among others. Taken together, these observations indicate that consumption of the TWD markedly enhanced colitis, delayed recovery from gut injury, and enhanced colon tumorigenesis likely via extensive changes in expression of immune-related genes in the colon mucosa.

## 1. Introduction

Colorectal cancer (CRC) is the third leading cause of cancer death and the third most commonly diagnosed cancer in men and women in the United States [1]. Epidemiological data suggest that risk of CRC is strongly linked to long-term inflammatory bowel diseases (IBD), of which the most common types are Crohn’s disease and ulcerative colitis (UC) [2,3,4]. The chronic intestinal inflammation that characterizes these conditions is now recognized as an important factor in CRC due to its involvement in the disruption of the same oncogenic pathways that are disrupted in CRC [5]. The mutation of genes involved in the maintenance of the intestinal mucosal barrier that protects the intestinal wall from bacterial invasion contributes to both Crohn’s and UC [6,7].

Dysfunction of the intestinal mucosal barrier leads to sustained damage of gut epithelial cells. This chronic injury to the gut triggers a compensatory immune response characterized by the up-regulation of cell proliferation and anti-apoptotic pathways that promote cell survival [8]. Various molecules are involved in the activation of such pathways, including transcription factors (e.g., NF-κB, and STAT3) and various inflammatory cytokines (e.g., IL-6 and TNF- α), which are normally secreted during an inflammatory response. The resulting compensatory cell regeneration results in increased rates of mitosis that, when chronically active, increase DNA mutation rates [8,9]. Not only can this process promote the disruption of oncogenic pathways, but it can also provide cancerous cells with a nurturing environment due to the increased availability of proliferative and survival signals. This notion is supported by an animal study by Tanaka et al. [10], in which a combined treatment of the chemical carcinogen azoxymethane (AOM) with the inflammatory agent dextran sodium sulfate (DSS) significantly increased intestinal tumorigenesis in CD-1 mice. The AOM + DSS model yields a consistent, reproducible colon cancer outcome that is well defined in terms of mouse strain and AOM dosage [11]. Moreover, this model recapitulates many of the molecular events that occur in spontaneous human colon cancer, such as β-catenin accumulation and K-ras mutations [12].

In recent decades, global incidence rates of colorectal cancer have been increasing, reportedly due to an increased prevalence of certain lifestyle trends, including decreased physical activity and consumption of “Westernized” diets [13]. The typical Western diet is characterized by increased intake of highly processed foods that contain excess levels of fat, sodium, and refined sugars and are generally low in some of the essential vitamins and minerals, as well as fiber [14,15,16]. Diet is one of the most influential factors affecting overall cancer incidence, with CRC being one of the most well-established examples. Evidence from epidemiological and pre-clinical animal studies supports the contention that the Western diet is positively associated with higher risk and incidence of CRC [14,16]. Yet, in the vast majority of pre-clinical animal studies designed to investigate molecular mechanisms of carcinogenesis or cancer prevention or to identify new strategies for dietary cancer prevention, researchers routinely employ standard basal diets that are generally balanced with respect to macro- and micronutrient levels to sustain animal health, such as the American Institute of Nutrition AIN93G/M diet formulations [17]. However, these standardized rodent diets are not relevant to most human nutrition patterns, especially for at-risk populations that frequently consume energy-dense, nutrient-poor foods. Other approaches to model Western nutrition in preclinical animal studies suffer from inconsistent reproducibility (e.g., the “cafeteria” diet) or insufficient consideration of diverse sources of dietary fat (e.g., the standardized diet-induced obesity diet).

In a series of studies over the past twenty years, Newmark and colleagues have employed a selective approach in modeling the Western diet, wherein specific components of the diet were modified to emulate typical U.S. intakes [18,19,20]. In their first study, Newmark et al. developed a “stress” diet, which was quite low in calcium and vitamin D_3_, and modestly reduced in phosphate compared to the reference diet AIN76; also, the stress diet contained 20% fat as corn oil (40% of calories) compared to only 5% (12% of calories) in the reference diet [18]. Rats and mice fed this stress diet for 12 weeks developed hyperplasia and hyperproliferation in the colon. Subsequent studies extended this stress diet to incorporate dietary components necessary for generation of methyl donor molecules (folic acid, methionine, choline, and vitamin B_12_) and determined that this new Western diet enhanced spontaneous tumor development in aged C57BL/6J mice, an effect that was reversed when calcium and vitamin D were added back to the stress diet [19,20]. Although this series of studies has demonstrated a role for dietary calcium and vitamin D_3_ in modulating spontaneous colon carcinogenesis in mice, the scope of the diet remained limited in that it did not consider possible contributions of the dietary fat source, carbohydrates or proteins and did not reflect typical Westernized nutrition patterns for other key micronutrients, such as sodium, selenium, or vitamins A or E. Commercial Western diets have also been developed for the study of obesity; these diets (referred to as diet-induced obesity, or DIO, diets) typically contain 45% or 60% of energy as fat and differ from the AIN93G diets primarily in their high lard and sucrose content [21]. Although these high fat diets effectively induce obesity in rodents [22], they are extreme in their sugar and fat compositions when compared to a typical Western dietary pattern and do not differ substantially from AIN93G diets in micronutrient content [21].

Importantly, none of the approaches described above for modeling typical Western nutrition appropriately considered the contribution of suboptimal micronutrient intakes in their disease models. To address this research gap, our research team developed the new total Western diet (TWD) for rodents with energy and nutrient profiles that emulate a typical Western diet using available U.S. nutrient intake survey data [23]. Briefly, the amount of each macro- and micronutrient in the AIN93G basal diet, a diet routinely used in cancer studies today, was adjusted to match 50th percentile intakes for Americans as reported in NHANES survey data. These mass amounts were then adjusted for caloric intake. Overall, the TWD is not necessarily extreme in the level of any given nutrient, but rather reflects the overall dietary pattern of the U.S. The TWD has fewer calories from protein and carbohydrate sources, and twice that from fat as compared to the AIN93G diet. The TWD contains more saturated and monounsaturated fats, less polyunsaturated fat, more complex carbohydrates, and twice the level of simple sugars. The TWD has less calcium, copper, folate, thiamine, and vitamins B_6_, B_12_, D, and E, but much more sodium. This newly devised diet that better represents typical U.S. nutrition is highly useful for studies employing animal models of human cancer. For example, results of a preliminary experiment in A/J mice initiated with AOM indicated that the TWD markedly enhanced development of preneoplastic ACF compared to the reference diet AIN93G [24]. Yet, the impact of a Western type diet on colon inflammation and subsequent colon tumorigenesis is not known. Given the importance of chronic colitis as a risk factor for development of inflammation-associated colorectal cancer (CAC), the overarching objective of this work was to test the hypothesis that consumption of the TWD would enhance colitis, delay recovery from gut injury, and promote colon tumorigenesis.

## 2. Materials and Methods

### 2.1. Chemicals and Reagents

Azoxymethane (AOM) was purchased from Sigma-Aldrich (St. Louis, MO; CAS No. 25843-45-2). Due to variable availability, reagent grade dextran sodium sulfate (DSS) was purchased from multiple sources, as indicated: Experiment A, mol. wt. 36-50 kDa, MP Biomedicals (via ThermoFisher Scientific, Waltham, MA); Experiment B-E, mol. wt. ~40 kDa, Alfa Aezar (via either VWR, Radnor, PA or ThermoFisher). All other chemicals used were of reagent grade and purchased from general laboratory suppliers.

### 2.2. Animals and Experimental Diets

The Utah State University Institutional Animal Care and Use Committee approved all procedures for the handling and treatment of mice used in this study (protocols 2114, 2262, 2404). Animals were housed in the Laboratory Animal Research Center (LARC) at Utah State University, which is an AAALAC approved facility. Mice were maintained in a specific pathogen-free vivarium at 18 to 23 °C with humidity at 50% and with a 12:12 h dark:light cycle. Mice were provided Bed-o’Cobs^®^ 1/4 bedding (Andersons, Cincinnati, OH) and housed in HEPA-filtered cages on a IVC Air Handling Solutions ventilated housing system (Tecniplast, Buguggiate, Italy). C57BL/6J mice were obtained from Jackson Laboratories (Bar Harbor, ME) at 3 or 4 weeks of age and acclimated to the vivarium for 1 week. All experimental diets were formulated by Envigo (Hackensack, NJ; formerly Harlan-Teklad) as outlined in Table 1. Diets were obtained as a single lot from the vendor and maintained at 4 °C for the duration of the study. For all experiments, fresh food was provided twice per week, and food consumption was monitored at each change (including accounting for spillage into the cage). Fresh water was provided weekly. Individual body weights were recorded once per week for all mice.

### 2.3. Experiment Designs

#### 2.3.1. Experiment A: AOM + DSS Model of CAC with the TWD

To determine the impact of a diet that emulates Western nutrient intakes on CAC, we employed the AOM + DSS mouse model using C57BL/6J mice. We hypothesized that exposure to the TWD would promote colon tumorigenesis, and that this effect would be mediated, at least in part, by the micronutrient faction of the diet. Experimental diets included (1) AIN93G, the standard basal diet routinely employed in rodent studies (AIN); (2) the total Western diet (TWD), as described in Hintze, et al. [23]; (3) a macronutrient-modified diet (MM), which was modeled after the 50th percentile of NHANES intakes (as with TWD), yet contained the same vitamin and mineral content as the AIN93G diet; (4) a vitamin- and mineral-modified diet (VMM), which was modeled after the 50th percentile of NHANES intakes for vitamins and minerals (as with TWD), yet contained the same macronutrient content as the AIN93G diet; and (5) a commercial diet-induced obesity diet (DIO) that contained 45% of energy as fat (primarily lard) and matched the AIN93G for micronutrient content. At 5 weeks of age, male C57BL/6J mice were assigned to diet groups using a random block design to equalize group body weight at the start of the study (*n* = 20 per diet group) (Figure S1). At 7 weeks of age, all mice were dosed *i.p.* with 10 mg/kg AOM prepared in sterile PBS and provided 1% (*w*/*v*) DSS, a colonic irritant, via their drinking water for 4 weeks followed by regular tap water for the remainder of the study. After 16 weeks, all mice were euthanized by CO_2_ asphyxiation and necropsied. Colons were isolated, flushed with PBS, cut open longitudinally, and stored at 4 °C in 70% (*v*/*v*) ethanol until further assessment.

#### 2.3.2. Experiment B: Restoration of Calcium and Vitamin D or Methyl Donor Micronutrients in TWD to Amounts in the AIN Diet

In this follow-up experiment, we sought to determine whether restoring calcium and vitamin D or methyl donor micronutrients that contribute to one-carbon metabolism to amounts present in the standard AIN93G diet would suppress TWD-enhanced colon tumorigenesis. We hypothesized that both modifications would reduce tumor multiplicity and tumor burden in mice fed TWD. Experimental diets included (1) AIN93G, the standard basal diet routinely employed in rodent studies (AIN); (2) the total Western diet (TWD), (3) the TWD with calcium and vitamin D concentrations restored to amounts in the AIN diet (TWD + CaVD); and (4) the TWD with the methyl donor micronutrients B_2_, B_6_, B_12_, choline and folate restored to amounts in the AIN diet (TWD + MD). At 5 weeks of age, male C57BL/6J mice were assigned to diet groups using a random block design to equalize group body weight at the start of the study (*n* = 30 per diet group) (Figure S1). On experiment day 21, all mice were dosed *i.p.* with 10 mg/kg AOM prepared in PBS and provided 1% (*w*/*v*) DSS, a colonic irritant, via their drinking water for 10 days followed by regular tap water for the remainder of the study. On experimental days 33 and 45, mice were temporarily placed in new cages blinded to treatment and then the disease activity index (DAI) was determined by summing four qualitative scores for symptoms of colitis, including stool consistency (0, solid; 1, slightly soft; 2, moderately soft; 3, soft; 4, moderately liquid or liquid), apparent blood in stool (0, none; 1, trace; 2, low amount; 3, moderate amount; 4, high amount), apparent rectal bleeding (0, none; 1, trace; 2, low amount; 3, moderate amount; 4, high amount) and weight loss since onset of DSS treatment. For scoring weight loss, the top of the scoring scale was determined to be 1 SD below the average change in body weight of the AIN control group for either the colitis or recovery time point (defined as *top*). Thus, weight loss scores were assigned as follows: 0, > *top*; 1, *top* to −5%; 2, −5% to −10%; 3, −10% to −20%; 4, <−20%. This scaling allows for estimation of the lack of weight gain as is appropriate for animals exposed to DSS during a period of rapid body weight increase. Each parameter score was summed to calculate the DAI with a maximum possible score of 20 for each individual animal. On day 112, all mice were euthanized by CO_2_ asphyxiation and necropsied. Colons were isolated, flushed with PBS, cut open longitudinally, and stored at 4 °C in 70% (*v*/*v*) ethanol until further assessment.

#### 2.3.3. Experiment C: Apc^Min/+^ Model of Intestinal Tumorigenesis with TWD and DIO Diets

To determine whether the tumor-promoting effects of TWD were restricted to the colon or to the AOM + DSS model of CAC, we next determined the impact of TWD using the *Apc^Min/+^* genetic mouse model of intestinal tumorigenesis. Heterozygotes of this strain spontaneously develop adenomas in the small intestine with high multiplicity, and inclusion of DSS in the treatment protocol markedly enhances tumorigenesis in the colon within a similar study time frame. Male C57BL/6J- *Apc^Min/+^* and female wild type C57BL/6J-*Apc*^+/+^ mice were obtained from Jackson Laboratories (Bar Harbor, ME) and bred to produce either wildtype or *Apc^Min/+^* offspring. At weaning, genotyping was performed by modification of PCR by Su et al. [25] using DNA that was isolated from ear tissue by a NaOH extraction method [26]. *Apc*-mutant (5′-TTCTGAGAAAGACAGAAGTTA-3′), *Apc*-wild-type (5′-GCCATCCCTTCACGTTAG-3′), and *Apc*-common (5′-TTCCACTTTGGCATAAGGC-3′) primers were used at concentrations of 250 nM, 42 nM, and 500 nM, respectively with AmpliTaq Gold^®^ 360 Master Mix (Life Technologies, Grand Island, NY). PCR was performed using a thermocyling protocol of 93 °C for 10 min; 40 cycles of 95 °C for 30 s, 55 °C for 30 s; 72 °C for 60 s; and a final extension of 72 °C for 3 min to generate amplicons 619 bp in length for both *Apc^Min/+^* and *Apc^+/+^* genotypes and amplicons of 313 bp in length for *Apc^Min/+^* genotype only. Amplicon size was verified by agarose gel electrophoresis. Genotyped offspring were randomly assigned to one of three experimental diet groups, AIN, TWD or DIO, with each group consisting of 20 male and 20 female *Apc^Min/+^* mice (Figure S1). At 5 weeks of age, half the mice in each dietary group were administered DSS at 1% (*w*/*v*) via drinking water for 10 days while the remaining half continued to receive normal tap water (final *n* = 10 males and 10 females for each diet/DSS subgroup). At 12 weeks of age, body composition was measured using an MRI scan (EchoMRI-700; EchoMRI, Houston, TX), and then the mice euthanized by CO_2_ asphyxiation and necropsied. The cecum, liver, kidneys, and spleen were weighed, immediately frozen in liquid nitrogen, and stored at −80 °C. Small intestines and colons were isolated, flushed with PBS, cut open longitudinally, and stored at 4 °C in 70% (*v*/*v*) ethanol until further assessment.

#### 2.3.4. Experiment D: Longitudinal Effects of TWD on Colitis and Recovery from Gut Injury

In this experiment, we sought to investigate the impact of TWD over the course of disease development as colitis progressed to colon tumorigenesis. We hypothesized that TWD exposure would exacerbate symptoms of colitis and delay recovery after cessation of DSS exposure. Male C57BL/6J mice were assigned to either AIN or TWD diet groups using a random block design to equalize group body weight at the start of the study (*n* = 48 per group) (Figure S1). On study day 21, mice were dosed *i.p.* with 10 mg/kg AOM and given 1% (*w*/*v*) DSS in the drinking water for 10 days. On study days 7, 32, and 45, the colitis DAI was determined for all mice as described above and two cages (*n* = 8 per group per time point) were randomly selected from each diet group for necropsy for histopathology (details below). All remaining mice (*n* = 21 to 23 per group) were necropsied on day 105. Prior to necropsy, body composition measurements were recorded by EchoMRI-700 (Houston, TX, USA). At necropsy, body, liver, kidney, spleen, and cecum weights were recorded. Colons were isolated, flushed with PBS, and cut open longitudinally. Randomly selected colon tissue samples were stored in 70% (*v*/*v*) ethanol and assessed within 24 h of collection, then transferred to 10% phosphate-buffered formalin for histopathology (details below). The remaining colon tissues were stored at 4 °C in 70% (*v*/*v*) ethanol until further assessment.

#### 2.3.5. Experiment E: Impact of TWD on DSS-induced Colitis and Expression of Immune- and Cancer-Related Genes in the Colon

Last, we aimed to determine the molecular mechanisms underlying the promoting effects of the TWD on colitis symptoms and the apparent delay in recovery following cessation of DSS-induced gut injury using a targeted immune gene expression profiling approach. Male C57BL/6J mice were assigned to either AIN or TWD diet groups using a random block design to equalize group body weight at the start of the study (*n* = 24 per group) (Figure S1). On study day 21, mice were given 1% (*w*/*v*) DSS in the drinking water for 10 days. On days 33 and 45, the colitis DAI was determined for all mice as described above. On study days 21, 33, and 45, two cages (*n* = 8 per group per time point) were randomly selected from each diet group for necropsy. Colons were isolated, flushed with PBS, and cut open longitudinally to expose the intestinal mucosa. Colon mucosa samples were collected by gentle scraping with a glass slide, immediately frozen in liquid nitrogen, and stored at −80 °C for transcript analysis.

### 2.4. Assessment of Tumors of the Colon or Small Intestine

To determine the effect of the experimental diets on tumorigenesis in the colon or the small intestine (Experiment C only), tissues were stained with 0.1% (*v*/*v*) methylene blue in PBS and analyzed under a dissecting microscope. The researcher was blinded to the sample identification at the time of assessment. Measurements of colon length and tumor volumes were obtained using a pair of electronic calipers. Colon tumors were identified and counted using the following criteria: (1) defined, generally round or oval shape, (2) defined, easily distinguishable edges, and (3) lighter color with respect to surrounding tissue. Tumor multiplicity was calculated as the number of tumors per mm colon for each tumor-bearing animal. Colon tumor volume was estimated using the formula volume = π/6(*L* × *W* × *D*), where *L* is the length, *W* is the width, and *D* is the depth of the tumor. Aberrant crypt foci (ACF) in the colon were also identified in using criteria described previously [27,28]. Briefly, ACF were characterized as crypts of larger size and often slit-shaped, with increased pericryptal area, and thickened layer of epithelial cells which sometimes resulted in greater staining around the crypt. Small intestine polyps were identified by the following criteria: (1) generally round with a floret or crater appearance, (2) dark and solid in color, (3) firm and inflexible. Tumor multiplicity was calculated as the number of tumors per mm small intestine for each tumor-bearing animal. Size of the small intestine lesions was estimated using the formula area = π/4(*L* × *W*), where *L* is the length and *W* is the width.

### 2.5. Histopathology and Immunohistochemistry

Colons selected for histopathology assessment were stored using the Swiss-roll method for 24 h in 10% (*v*/*v*) formalin and then transferred to 70% (*v*/*v*) ethanol prior to embedding in paraffin. Microscopy slides were prepared from 5 μm sections of the paraffin block and stained with hematoxylin and eosin per standard protocol. These samples then were submitted to the Utah Veterinary Diagnostic Laboratory for histopathological analysis by a board-certified pathologist. Tissues were blinded as to treatment and then assessed for inflammation and mucosal injury using a grading scheme previously described [29,30]. Sections were scored on a 0 to 4-point scale for three parameters: inflammation/cellular infiltration, epithelial regeneration and crypt damage. For inflammation, severity and depth were separately assessed, combined into one score, and then multiplied by a factor reflecting the percentage of the colon involved (1, 0%–25%; 2, 26%–50%; 3, 51%–75%; and 4, 76%–100%) to obtain the overall score. Assessment of the epithelium was evaluated by averaging the severity of crypt loss or ulceration over at least 20 high power fields (400×; 4.74 mm^2^). For mucosal injury, crypt damage and regeneration were also summed and multiplied by a factor reflecting the percentage of the colon involved (as outlined above) to obtain an overall score.

Selected formalin-fixed paraffin-embedded sections of tissues from Experiment A were subject to standard immunohistochemistry protocols recommended by the antibody supplier for detection of beta-catenin and Ki67. Briefly, tissue sections (5 μm thick) were cut and antigen retrieval was achieved by heating the sections in 10 mM sodium citrate with 0.05% (*v*/*v*) Tween-20 at pH 6.0 for 10 min in a pressure cooker. Sections were then incubated as follows: 30 min with Background Punisher blocking reagent (Biocare Medical); 30 min with primary antibodies for either anti-beta catenin (1:500 dilution; biomarker of activation of the Wnt signaling pathway) or anti-Ki67 (1:50 dilution; biomarker of cell proliferation); 30 min with rabbit-on-rodent HRP-polymer; 15 min with Warp Red^TM^ chromagen (Biocare Medical); and 5 min hematoxylin counterstain.

### 2.6. Analysis of Transcript Abundance Data for Colon Mucosa

Gene expression analyses of colon mucosa samples obtained from Experiment E were performed largely as described previously [31], with some modifications outlined below. Total RNA was extracted from the total colon mucosa with TriReagent (Sigma Aldrich, St. Louis, MO, USA) per manufacturer’s instructions. Resultant RNA was further purified and genomic DNA removed by RNeasy Mini Kit with DNase treatment (Qiagen, Valencia, CA, USA). RNA was dissolved in nuclease-free water and quantified using Qubit (Thermo Fisher), and RNA purity was verified by determining 260/280 and 260/230 absorbance ratios by NanoDrop Spectrophotometer (NanoDrop 1000, Wilmington, DE). RNA (*n* = 6–8/group) was analyzed using the nCounter Mouse PanCancer Immune Profiling Panel (NanoString Technologies, Seattle, WA, USA), which includes 750 immune-related genes, 20 housekeeping genes, 6 positive controls and 8 negative controls. Assays were performed and quantified on the nCounter MAX system, sample preparation station, and digital analyzer (NanoString Technologies) according to the manufacturer’s instructions. Briefly, reporter and capture probes were hybridized to target analytes for 16 h at 65 °C. After hybridization, samples were washed to remove excess probes. Then, the purified target-probe complexes were aligned and immobilized onto the nCounter cartridge, and the transcripts were counted via detection of the fluorescent barcodes within the reporter probe. Probe annotations are provided in File S1.

Raw gene expression data were analyzed using NanoString’s software nSolver v3.0.22 with the Advanced Analysis Module v2.0. Background subtraction was performed using the eight included negative controls included with the module. Genes with counts below a threshold of 2σ of the mean background signal were excluded from subsequent analysis. Data normalization was performed on background-subtracted samples using internal positive controls and selected housekeeping genes that were identified with the geNorm algorithm (https://genorm.cmgg.be/). Ratios of transcript count data for all diets and time points were generated using the AIN diet group at the Pre-DSS time point (AIN/Pre-DSS) as a reference. Ratios were then log_2_ transformed for downstream analysis and data presentation.

Differential gene expression analyses were performed using the nSolver Advanced Analysis Module, which employs several multivariate linear regression models (mixture negative binomial, simplified negative binomial, or log-linear model) to identify significant genes. Resulting *p* values were adjusted using the Benjamini–Hochberg (BH) method to control the false discovery rate. A statistically significant difference in gene expression was defined as two-fold change in expression (log_2_ > 1 or < −1) with BH *q* < 0.05. Nine pairwise comparisons were determined a priori, as follows: (1) colitis vs. pre-DSS fed AIN; (2) recovery vs. pre-DSS fed AIN; (3) recovery vs. colitis fed AIN; (4) colitis vs. pre-DSS fed TWD; (5) recovery vs. pre-DSS fed TWD; (6) recovery vs. colitis fed TWD; (7) TWD vs. AIN at pre-DSS time point; (8) TWD vs. AIN at colitis time point; and (9) TWD vs. AIN at recovery time point. Outputs from nSolver differential expression analysis are provided in File S2. Venn diagrams of significant differentially expressed genes were generated using BioVenn [32].

To assess the impact of experimental diets on annotated gene sets, global and directed significance scores were calculated for each pathway at each time point. The global significance score for each gene set was calculated as the square root of the mean squared *t*-statistic of genes, as determined by the differential gene expression analyses. The global score estimates the cumulative evidence for the differential expression of genes in a pathway. The directed significance score was also calculated by considering the sign of the *t*-statistics. Directed significance scores near zero indicate that a pathway may have many highly regulated genes, but no apparent tendency for those genes to be over- or under-expressed collectively. As a complementary method for comparing pathways and discriminating between experimental groups, pathway Z scores were calculated as the Z-scaled first principal component of the pathway genes’ normalized expression. Files S3-S4 provide summary tables for all significance and pathway Z scores, respectively. ClustVis [33] was used to perform unsupervised hierarchical cluster analyses (HCC) and principal components analyses (PCA) using log_2_ transcript count data for differentially expressed genes. Finally, Spearman rank correlations were performed to examine overall patterns in the gene expression profiles using the pathway *Z* score compared to the observed DAI at the colitis and recovery time points. A significant correlation was inferred when *ρ* > 0.5 or < −0.5 and *p* < 0.05. Network analyses for interactions among significant genes were performed using STRING database version 10.5 (http://string-db.org/), which curates both experimental and predicted gene interactions. Only interactions among significant DEGs were considered with the confidence level for associations set at ≥ 0.9. Clusters were identified using the Markov Cluster (MCL) algorithm with inflation parameter of 1.5. Networks generated by STRING were visualized with Cytoscape v. 3.5. File S5 provides data for STRING-db networks and the predicted clusters. The PathView module in nSolver was used to overlay differential expression analyses results with KEGG pathways for visualization of differentially expressed genes in context of relevant biological pathways. To determine which biological processes were associated with DEGs for either AIN or TWD at either colitis or recovery time points, gene ontology enrichment was performed using the complete set of detected genes as the background list (Metascape, [34]). A significant enrichment in biological process terms associated with lists if DEGs was inferred when FDR *q* < 0.05 and enrichment was at minimum 1.5. Redundant terms were clustered by similarity (kappa score > 0.3), and the term with the most significant *q*-value within a cluster was used to identify that cluster. Complete gene ontology results from Metascape are provided in File S6.

The NanoString nSolver Advanced Analysis software employs the method described by Danaher [35] to measure the abundance of various immune cell populations using marker genes that are expressed stably and specifically in particular cell types. Cell type scores were calculated as the average log-scale normalized expression of their characteristic genes. Relative cell type measurements were based on the total population of infiltrating lymphocytes, which is useful in a sample of heterogenous mix of cell types. While this analysis did not convey information on the absolute number of immune cells in any one sample, comparisons were made within each cell type across test diets and time points. Only cell types that exceeded the quality control analysis for correlation of marker gene expression are reported herein, including the following cell types and their marker genes: B-cells (*Ms4a1, Tnfrsf17*), T-cells (*Cd3d, Cd3e, Cd3g, Cd6, Sh2d1a*), CD8 T-cells (*Cd8a, Cd8b1*), dendritic cells (*Ccl2, Cd209e, Hsd11b1*), neutrophils (*Csf3r, Fcgr4*), and CD45 (*Ptrpc*). File S7 provides both the raw and relative cell type scores calculated by nSolver software.

### 2.7. General Data Analysis

Statistical analyses for tumor incidence were performed using the Fisher’s exact test, followed by a Bonferroni adjustment to correct for multiple testing (Prism v. 8, GraphPad Software, San Diego, CA). Other data were analyzed using a linear mixed model for main effects of diet, time point, and/or sex (experiment C only) with cage as a nested, random factor using the restricted maximum likelihood (REML) estimation and the Tukey HSD post-hoc test for multiple comparisons (JMP v. 12, SAS Institute, Cary, NC). Suspected outliers were verified using the robust outlier test (ROUT) with a conservative *Q* value of 1% (Prism), meaning that there was ≤1% chance of excluding a data point as an outlier in error. Data that did not meet the equal variance assumption were log_10_ or sqrt transformed. For data that were not normally distributed or for which a transformation did not equalize variance, the non-parametric Steel–Dwass test was used (JMP). A significant effect of the test variable was inferred when the adjusted *p* value was < 0.05. Energy intakes were assessed on a per cage basis.

## 3. Results

### 3.1. Experiment A: AOM + DSS Model of CAC with the TWD

**Figure 1:**
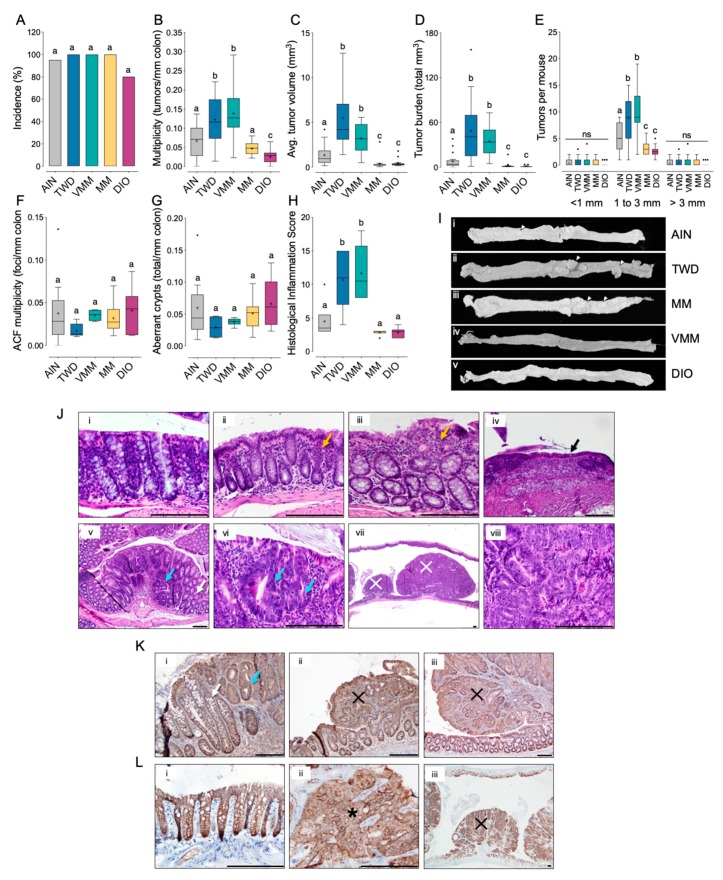
Consumption of the TWD or the vitamin- and mineral-modified diet promoted colon tumorigenesis in a mouse model of inflammation-associated colorectal cancer (Experiment A). C57BL/6J male mice were fed the standard AIN93G diet (AIN), the total Western diet (TWD), the vitamin- and mineral-modified diet (VMM), the macronutrient-modified diet (MM) or the 45% fat diet-induced obesity diet (DIO). (**A**) Incidence of colon tumors shown as the percent of mice with tumors. (**B–H**) Colon tumor multiplicity (**B**), average tumor volume (**C**), tumor burden (**D**), tumor multiplicity segregated by tumor size (**E**), aberrant crypt foci (ACF) multiplicity (**F**), number of aberrant crypts (**G**), and histological inflammation score (**H**) data are shown as Tukey box plots (box, 25th to 75th percentiles; whiskers, 1.5 IQR; +, mean) (*n* = 19 to 20 for tumor endpoints; *n* = 6 for histological inflammation score). Different letters indicate that diet treatment groups are significantly different (*p* < 0.05) as determined as determined by the appropriate linear mixed model or nonparametric test as outlined in the Materials and Methods. For multiplicity data segregated by tumor size (**E**), data were stratified by tumor size prior to statistical analyses. (**I**) Gross pathology images depicting colon tissues representative of each diet treatment group as follows: i, AIN, ii, TWD; iii, VMM; iv, MM, and v, DIO. White arrows indicate large colon tumors easily visible via low magnification light microscopy. (**J**) H&E staining of colon tissues depicting different stages of disease, as follows: i, normal (400×); ii, mild inflammation (infiltration of neutrophils, lymphocytes and plasma cells in the submucosa, yellow arrow) (400×); iii, moderate inflammation (infiltration of neutrophils, lymphocytes and plasma cells in the submucosa, yellow arrow) (400×); iv, healed mucosal ulcer with complete loss of colonic glands and neutrophilic and lymphoplasmacytic inflammation (black arrow) (200×); v, gastrointestinal intraepithelial neoplasia (GIN, blue arrow) alongside normal tissue (white arrow) (100×); vi, GIN (blue arrow) (400×); vii, high grade adenoma (**✕**) (20×); viii, high grade adenoma (**✕**) (400×). (**K**) Expression of ki-67 in colon tissues: (i) normal (white arrow) and GIN (blue arrow) (200×), (ii) high grade adenoma (**✕**) (200×); (iii) high grade adenoma (**✕**) (100×). (**L**) Expression of beta catenin colon tissues: (i) normal colon (400×); (ii), adenoma (*) (400×); (iii) high-grade adenoma (**✕**) (40×). Scale bars = 200 μm.

In this first experiment, we tested the TWD in comparison to the standard AIN93G diet designed to sustain rodent health and the 45% fat DIO diet commonly used to induce obesity in C57BL/6J male mice. To determine the contribution of macro- or micronutrients on cancer outcomes, we also split the TWD into its macro- (MM diet) or micronutrient (VMM) fractions. As expected with this initiation protocol, nearly all animals acquired colon cancer. When correcting for multiple testing, no significant differences were noted among the diet groups for colon tumor incidence (Figure 1A). 

**Figure 2:**
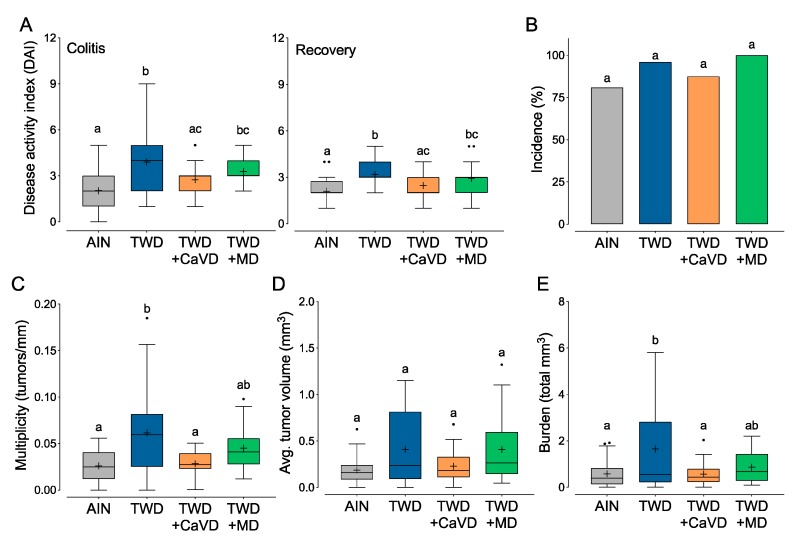
Restoration of calcium and vitamin D to amounts in the AIN diet suppressed colitis symptoms and inflammation-associated colon tumorigenesis in C57BL/6J male mice fed TWD (Experiment B). Diet groups included the standard AIN93G diet (AIN), the total Western diet (TWD), the TWD with calcium and vitamin D restored to AIN amounts (TWD + CaVD) or the TWD with methyl donor micronutrients B_2_, B_6_, B_12_, folate, and choline restored to AIN amounts (TWD + MD). (**A**) Colitis disease activity index (DAI) score (*n* = 29 to 32) at colitis and recovery time points. (**B–E**) Colon tumor outcomes include incidence (**B**), tumor multiplicity (**C**), average tumor volume per mouse (**D**), and burden as total tumor volume (**E**) (*n* = 23 to 26). Data in all panels (except B) are shown as Tukey box plots (box, 25th to 75th percentiles; whiskers, 1.5 IQR; +, mean). Different letters indicate that treatment groups are significantly different (*p* < 0.05) as determined by the appropriate linear mixed model or nonparametric test as outlined in the Materials and Methods.

On the other hand, striking significant effects of experimental diet on tumor multiplicity and size were observed. Mice provided either the TWD or VMM diets had nearly twice as many tumors as their AIN-fed counterparts (*p* = 0.0338 or *p* = 0.0024, respectively), and about 3-times the number of tumors as animals provided the MM or DIO diets (*p* < 0.0001) (Figure 2B). However, tumor multiplicity was not significantly different in animals fed the MM or DIO diets compared to the AIN93G group. A very similar pattern for tumor volume was also evident, as the average tumor size in mice provided TWD or VMM diets was about 3.6- to 2.5-fold higher than in mice fed AIN diet (*p* < 0.005) (Figure 1C). An even more pronounced, 10-fold increase in average tumor size was observed when comparing the TWD and VMM groups to mice fed either the MM or DIO diets (*p* < 0.0001). Moreover, the tumor burden (defined as the total volume of tumor tissue) in mice fed TWD and VMM was markedly higher compared to their counterparts fed AIN (*p* ≤ 0.0001). These differences in CAC outcome between dietary treatments were apparent in tumors sized between 1 and 3 mm but not larger (< 3 mm) or smaller (> 1 mm) tumors (Figure 1E). Thus, in terms of overall CRC phenotype, mice fed the VMM diets were most similar to mice fed the TWD, while MM fed mice were most similar to mice fed AIN and DIO diets. To determine whether mice fed MM or DIO diets acquired a large number of preneoplastic lesions that failed to progress to tumors, we counted the number of aberrant crypt foci (ACF) and the average number of aberrant crypts (Figure 1F,G). Although no significant differences among diet groups were observed, the low variance evident in mice fed the TWD and VMM likely reflects that many of the preneoplastic lesions in these diet groups progressed to form colon tumors. Not surprisingly, dietary treatment resulted in a pattern of gut inflammation that corresponded to CAC outcome. Mice fed the TWD and VMM diets had increased histological inflammation scores relative to all other treatments (*p* < 0.05) (Figure 1H).

Tumors were readily apparent via gross examination of colon tissues, with very large tumors evident in mice fed TWD or VMM diets (Figure 1G). Histologic assessment of H&E-stained tissue sections revealed evidence of mild multifocal lymphoplasmacytic colitis and presence of colon adenomas (low and high grade) in some tissues from AIN-fed mice, as expected for the AOM + DSS model of CAC. In mice fed TWD or VMM diets, histologic analysis typically revealed multiple high-grade tubular colonic adenomas, gastrointestinal intraepithelial neoplasia (GIN), and moderate lymphoplasmacytic multifocal colitis. In mice fed DIO or MM diets, minimal or mild multifocal lymphoplasmacytic colitis was noted with no tumors observed microscopically in the tissues examined. Example tissue sections stained with H&E, probed for beta-catenin expression, or probed for Ki67 expression that typify these histological features (e.g., colitis, GIN and adenoma) are provided in Figure 1J–L.

During this study, we monitored food and energy intake and body weight gain. We previously reported the impacts of these experimental diets on body weight, body composition, glucose tolerance and other measures related to metabolic syndrome for sham-initiated mice [see results in 36]. Energy intake in AOM-initiated mice was not significantly different for mice fed AIN, TWD, VMM or MM diets, whereas DIO-fed mice consumed substantially more food energy (Figure S2A). This excess energy intake translated to a substantial increase in average body weight for DIO-fed mice at the end of the study. Similarly, mean body weight was also increased for mice fed MM diet (Figure S2B). However, mice fed TWD and VMM did not gain more weight, reflecting their overall normalized energy intake. At the end of the study, colon length was not notably correlated with evidence of inflammation or tumorigenesis, as the only noted difference was between the VMM and DIO diet groups (Figure S2D).

### 3.2. Experiment B: Restoration of Calcium and Vitamin D or Methyl Donor Micronutrients in TWD to Amounts in the AIN Diet

To determine which micronutrients might be responsible for the differential CAC response between mice fed the AIN and TWD in the previous experiment, modifications were made to the micronutrient portion of the TWD. We hypothesized, based on previous work from Newmark and colleagues [18,19,20], that suboptimal concentrations of calcium and vitamin D or methyl donors in the TWD were responsible for enhanced colon tumorigenesis. Therefore, experimental diets were formulated that included: the TWD + dietary calcium and vitamin D at the same amount as AIN93G diet (TWD + CaVD) and TWD + dietary methyl donors at the same amount as AIN93G diet (TWD + MD, Figure S1). During active colitis, mice fed TWD had nearly 2-fold higher DAI score (*p* < 0.0001) than those fed AIN (Figure 2A), and this effect persisted to recovery (Figure 2B). Restoration of calcium and vitamin D to amounts present in the AIN diet appeared to suppress the DAI during colitis and recovery such that symptoms were not different from the AIN diet group (*p* = 0.2313). Alternatively, colitis symptoms were not significantly changed in mice fed TWD + MD as compared to either TWD or AIN diets (Figure 2A). Colon tumor incidence was not different among any of the test diets. However, as observed in the prior experiment, consumption of the TWD significantly increased colon tumor multiplicity (*p* = 0.0099) and burden (*p* = 0.0264); although tumor volume was greater in TWD-fed mice, this response was highly variable and not significantly different from AIN-fed animals. Of note, the tumor response in mice fed the TWD + CaVD diet mirrored earlier observations for DAI in this model of CAC, with significant decreases in tumor multiplicity (*p* = 0.0314) and tumor burden (*p* = 0.0306) evident compared to TWD-fed mice (Figure 2C–E). These observations suggest that addition of calcium and vitamin D back to the TWD was sufficient to rescue the carcinogenic phenotype of the TWD. Conversely, supplementation of the TWD with methyl donor micronutrients had a more moderate effect on these endpoints, as the tumor response in mice fed TWD + MD was not significantly different compared to the either the AIN or TWD treatments for any of these measures (Figure 2C–E).

Mice fed the TWD + CaVD diet consumed more energy than did their counterparts fed AIN or TWD (*p* < 0.05), but not more than those fed TWD + MD (Figure S3A). However, the only apparent difference in final body weight was noted when comparing AIN-fed mice to those provided TWD + CaVD (Figure S3B). In this experiment, the body composition of mice in any group provided the TWD basal diet was significantly shifted toward fat as compared to those fed the AIN diet, which was entirely attributed to a gain in fat mass (Figure S3C) as opposed to a loss of lean mass (Figure S3E). Cecum weights did not differ among mice fed the different TWD diets, however, mice fed the AIN diet had larger ceca than the TWD fed mice (Figure S3G). Dietary treatment did not affect colon length at necropsy (Figure S3H).

### 3.3. Experiment C: Apc^Min/+^ Model of Colorectal Carcinogenesis with TWD and DIO Diets

**Figure 3:**
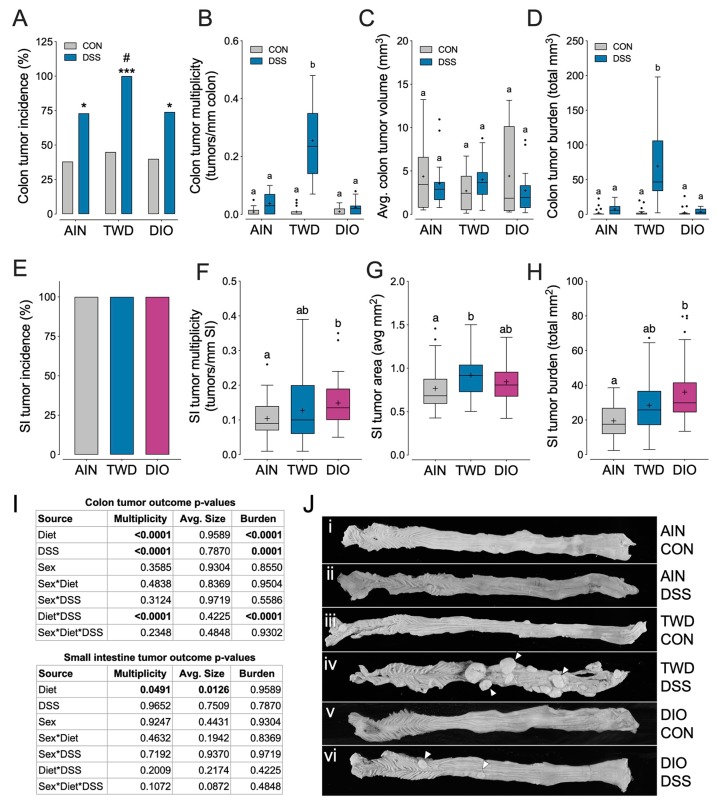
TWD promotes inflammation-associated colon tumorigenesis in *Apc^Min/+^* mice, but does not markedly alter small intestine tumor development (Experiment C). *Apc^Min/+^* female and male mice were fed a standard diet (AIN), the total Western diet (TWD) or the 45% fat diet-induced obesity diet (DIO) for 12 weeks. Half the mice were provided 1% DSS via drinking water for two weeks. (**A**) Incidence of colon tumors shown as the percent of mice with tumors. * *p* < 0.05, *** *p* < 0.001 for comparisons of DSS to CON group within each diet and # *p* < 0.05 for comparison across diet groups within CON or DSS cohorts as determined by the Chi-square test. (**B–D**) Data shown include colon tumor multiplicity (**B**), average size of colon tumors (as volume) (**C**) and colon tumor burden (as total volume) (**D**). (**E**) Incidence of small intestine tumors shown as the percent of mice with tumors. (**F–H**) Data shown indicate small intestine (SI) tumorigenesis endpoints for diet and DSS treatment, including SI tumor multiplicity (**F**), average size of SI tumors (as area) (**G**), and burden of SI tumors (as total area) (**H**). Data in B–D and F–H are shown as Tukey box plots (box, 25th to 75th percentiles; whiskers, 1.5 IQR; +, mean) (*n* = 18 to 22). Different letters indicate that groups are significantly different (*p* < 0.05) as determined by the appropriate linear mixed model or nonparametric test. For SI tumor outcomes, comparisons were made by diet group only as no main effect of or interaction with DSS was observed. (**I**) Main effect *p*-values for diet, DSS treatment, sex and all possible interactions are indicated to the right of charts for colon tumor data (top) or SI data (bottom). (**J**) Gross pathology images depicting colon tissues representative of each diet treatment group as follows: i, AIN and no DSS; ii, AIN+DSS; iii, TWD and no DSS; iv, TWD+DSS; v, DIO and no DSS; vi, DIO+DSS. White arrows indicate large colon tumors easily visible via low magnification light microscopy.

In order to determine if the tumorigenesis response to TWD was limited to the AOM + DSS model, *Apc^Min/+^* mice were fed the AIN, TWD or DIO diets with and without DSS. For all dietary treatments, addition of DSS to drinking water resulted in a higher incidence of colon tumors (*p* < 0.05). Of note, for mice provided DSS, those fed TWD had significantly greater incidence compared to their counterparts fed either AIN or DIO diets (*p* = 0.0163 and 0.0193, respectively). For colon tumor multiplicity and burden, the experimental diet and DSS treatments were significant factors (main effect *p* < 0.0001) in the mixed model, and a significant interaction was also noted (*p* < 0.0001) as clearly demonstrated by the very robust tumorigenesis response observed in mice fed TWD and provided DSS to trigger inflammation (Figure 3B,D). Average tumor multiplicity in TWD-fed, DSS-treated *Apc^Min/+^* mice was nearly 70- to 100-fold greater (2.55 ± 0.032 tumors/mm) compared to their counterparts fed AIN (0.038 ± 0.0072 tumors/mm) or DIO (0.024 ± 0.0053 tumors/mm) (*p* = 0.0018 and =0.0002, respectively). A similar pattern was noted for tumor burden (Figure 3D), but interestingly, tumor size was not affected by diet or DSS treatment (Figure 3C). Mouse sex was not a significant factor for any colon tumor parameter assessed. The small intestine tumor phenotype was marginally affected by dietary treatment (main effect *p* = 0.0491), but not by DSS or sex. Incidence of small intestine tumors was 100%, as anticipated for this model (Figure 3E). Mice fed the DIO diet had increased tumor multiplicity and burden relative to AIN but not TWD fed mice (Figure 3F,H), although tumor size was marginally greater in mice fed TWD compared to their counterparts (Figure 3G). Representative colons from animals fed either AIN, TWD, or DIO with or without DSS treatment are presented in Figure 3J. Note that large colon tumors were easily visible via low magnification light microscopy in mice fed the TWD, whereas few tumors were evident in mice fed AIN or DIO diets and provided DSS, or for mice provided plain drinking water.

Dietary treatment had no effect on energy intake, although male mice fed TWD had a marginally higher average final body weight compared to their counterparts fed AIN or DIO diets (Figure S4). Fat and lean mass distribution was also affected by dietary treatment. Female TWD-fed mice had a higher relative fat mass and corresponding lower relative lean mass compared to AIN- or DIO-fed females, whereas male DIO-fed mice had higher relative fat mass and corresponding lower lean mass (Figure S4C,D). Similar to the previous experiment, the cecum weight was greater in AIN-fed mice compared to those provided TWD, but not the DIO diet (Figure S4E). Although colon length at necropsy was largely unaffected by dietary or DSS treatment, a main effect of sex was noted (*p* = 0.0179) with colon length slightly longer in males (69.2 ± 0.99 mm) than females (65.7 ± 0.79 mm) (Figure S4F). An interaction between diet and DSS treatment was also noted for colon length, driven apparently by a significant difference in mice fed TWD with plain water compared to mice fed DIO with DSS, although this comparison was not of primary interest (Figure S4F).

### 3.4. Experiment D: Longitudinal Effects of TWD on Colitis and Recovery from Gut Injury

**Figure 4:**
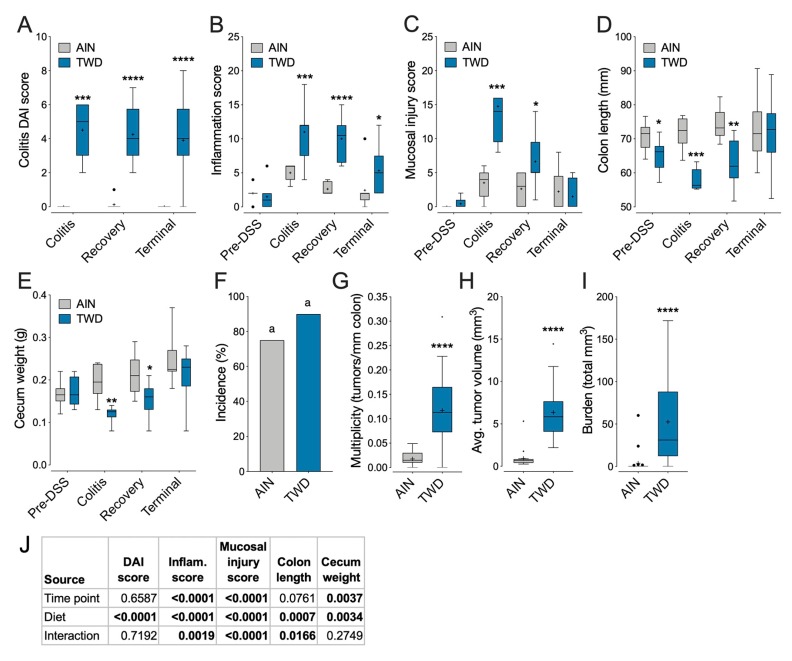
Consumption of TWD enhanced symptoms of colitis, colon inflammation and mucosa injury while also delaying recovery from gut injury caused by 1% DSS treatment (Experiment D). C57BL6/J male mice were fed AIN or TWD diets and provided DSS for 10 days to trigger colon inflammation. (**A**) DAI score (*n* = 8 at colitis and recovery, *n* = 20-23 at terminal time point). (**B**,**C**) Histopathology inflammation score (**B**) or mucosal injury score (*n* = 6 to 9). (**D**,**E**) Colon length (**D**) and cecum weight (**E**) (*n* = 7-8 for pre-DSS, colitis and recovery; *n* = 20-23 at terminal time point). (**F–I**) Colon tumorigenesis outcomes, including incidence (**F**), multiplicity (**G**), average tumor volume per mouse (**H**) and burden as the total tumor volume (**I**). Data in all panels (except F) are shown as Tukey box plots (box, 25th to 75th percentiles; whiskers, 1.5 IQR; +, mean). * *p* < 0.05; ** *p* < 0.01; *** *p* < 0.001; **** *p* < 0.0001 for TWD compared to AIN diet as determined by linear mixed model analyses for diet by time point with LS Means Student’s *t-*test post-hoc test between AIN and TWD diet groups. (**J**) Main effect *p*-values for diet, DSS treatment, sex and all possible interactions are shown for DAI score, inflammation score, mucosal injury score, colon length and cecum weight.

Next, we performed a longitudinal study comparing the colitis and carcinogenesis phenotype in mice fed either the TWD or AIN diets before DSS treatment, during active colitis, during recovery, and at the study end using the AOM + DSS model. Exposure to the TWD markedly increased symptoms of colitis throughout the study (Figure 4A). The histologic inflammation score was also increased for all time points after DSS treatment in mice fed the TWD, while the mucosal injury score was elevated during colitis and into recovery after cessation of DSS (Figure 4B,C) (*P* > 0.05, Figure 4B). We also observed that the colon length was significantly shorter for mice fed TWD prior to DSS treatment, through colitis and into recovery (Figure 4D); however, by the end of the study, the effect of diet on colon length was not evident. Furthermore, cecum weight was lower in the TWD fed mice at the colitis and recovery timepoints (Figure 4E). At the study end, tumor outcomes were largely similar to observations from prior experiments, with TWD markedly increasing tumor multiplicity, average tumor volume and tumor burden (*p* < 0.0001) relative to AIN-fed mice.

**Figure 5:**
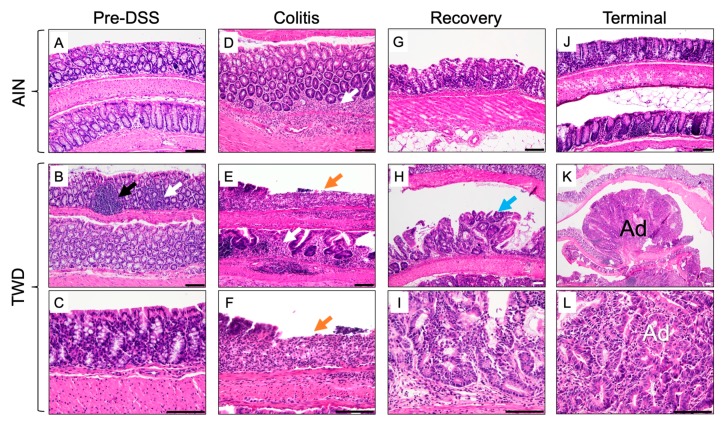
Histology of colonic tissues during the course of disease development. Representative images depicting H&E staining of colon tissues in mice fed AIN or TWD diets obtained prior to DSS treatment (**A–C**), during active colitis (**D–F**), during recovery from gut injury (**G–I**) and at the study end (**J–L**). Descriptions of tissue histology and image magnification: (**A**) normal colon (200×); (**B**) normal colon with occasional mild multifocal lymphocytic inflammation (white arrow) and gut-associated lymphoid tissue (black arrow) (200×); (**C**) normal colon (200×); (**D**) neutrophilic and lymphoplasmacytic colitis with inflammation in the mucosa and submucosa (white arrow) (400×); (**E,F**) neutrophilic and lymphoplasmacytic colitis with inflammation of the mucosa and submucosa (white arrow), mucosal ulceration (orange arrow), and loss of glands (E, 200×; F, 400×); (**G**) colon with mild lymphoplasmacytic and neutrophilic mucosal inflammation (200×); (**H,I**) Gastrointestinal intraepithelial neoplasia (blue arrow) (H, 100x; I, 400×); (**J**) normal colonic mucosa (200×); (**K**) colon adenoma (**A**) at low magnification (40×); and (**L**) colon adenoma (Ad) at high magnification (400×). Scale bar = 100 μm.

Representative histologic images depicting H&E-stained colon tissues at each disease stage are provided in Figure 5. Prior to DSS treatment, the colonic epithelium of AIN- and TWD-fed mice was intact with only mild multifocal inflammation evident in the mucosa in some mice. Following AOM/DSS initiation during active colitis, the colonic mucosa of AIN-fed mice was typified by mild to moderate multifocal neutrophilic infiltration (Figure 5D), whereas moderate to severe inflammation, extensive crypt and surface epithelium loss, and little tissue regeneration were evident in the colon tissues of TWD-fed mice (Figure 5E,F). Fourteen days later during recovery from gut injury, colons of AIN-fed mice showed signs of regeneration with only mild-to-moderate inflammation present (Figure 5G). However, for mice fed TWD, tissue regeneration was poor, with evidence of gastrointestinal intraepithelial neoplasia and persistent extensive neutrophilic inflammation (Figure 5H,I). By the study end, tissue of mice fed AIN was largely similar to that prior to the DSS exposure, with mild multifocal inflammation (Figure 5J) (no adenomas were observed in the mice subject to histopathology in AIN-fed mice at recovery, although some were seen macroscopically in other mice belonging to this group). Alternatively, by the end of the study, all TWD-fed mice that were examined by histology were had extensive neoplasia, including gastrointestinal intraepithelial neoplasia, adenoma, and high-grade tubular adenoma and/or adenocarcinoma (Figure 5K,L).

Lastly, in this experiment, dietary treatment did not significantly affect energy intake, body weight, or body composition (Figure S5). 

### 3.5. Experiment E: Immune- and Cancer-Related Gene Expression and cell Types in Colon Mucosa of TWD-fed Mice

**Figure 6:**
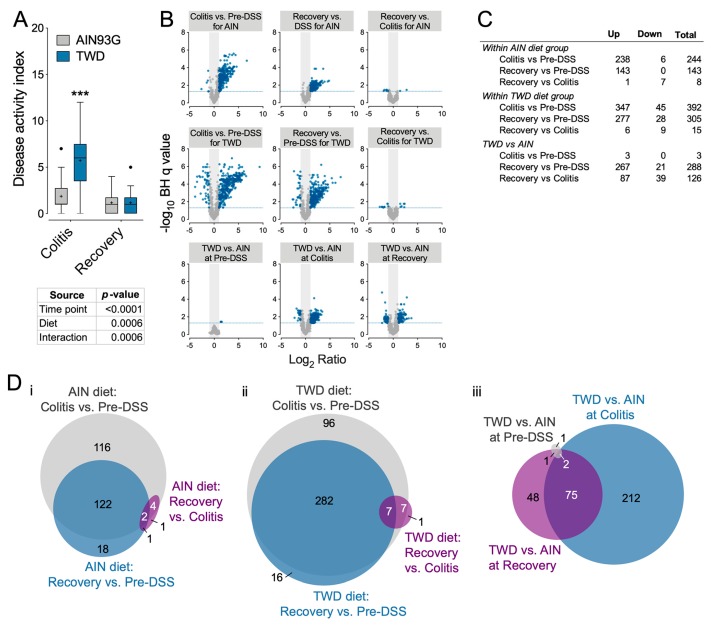
Expression of immune and cancer pathway genes in colon tissues was impacted to a greater extent in mice fed TWD as compared to those fed AIN during colitis and recovery (Experiment E). (**A**) Disease activity index (DAI) for mice fed AIN or TWD and given DSS to induce gut inflammation at colitis and recovery time points. (**B**) Volcano plots depicting immune-related mRNA expression as measured using the NanoString Mouse PanCancer Immune panel. Values shown are log_2_ ratios for each comparison indicated plotted against the -log_10_ of the Benjamini–Hochberg FDR *q* value. A significant difference in gene expression was inferred for genes with BH *q* value < 0.05 and log_2_ ratio > 1 or < −1. (**C**) Number of significant differentially expressed genes across time points within each diet or comparing AIN vs. TWD at each time point. (**D**) Proportional Venn diagrams depicting the number of genes identified as differentially expressed in colon tissues of mice fed either AIN or TWD diets. Comparisons were made by time point for AIN diet (i), by time point for TWD diet (ii), or by diet at each time point (iii).

To determine how exposure to the TWD exacerbates colitis and, subsequently, promotes colon tumorigenesis in mice, we determined the dynamic expression of 750 immune- and cancer-related genes in colon mucosa samples obtained prior to DSS insult, during active colitis and during recovery from colitis from mice fed either AIN or TWD. In Experiment E, DSS-induced colitis symptoms were markedly worse in mice fed TWD compared to those fed AIN (Figure 6A). However, this effect did not persist to the recovery time point as was previously observed for animals initiated with both AOM and DSS, suggesting that the unresolved increase in inflammation was due to the interaction of the carcinogen and irritant. Comparison of gene expression in colon tissues from AIN-fed mice at colitis compared to pre-DSS time point revealed 244 differentially expressed genes (DEGs), while a substantially larger number of DEGs were noted in TWD-fed mice (Figure 6B,C), including 212 that were uniquely impacted by TWD (Figure 6D). Immune-related gene response at the recovery time point was notably diminished in AIN-fed mice as compared to pre-DSS, with only 143 DEGs identified and the bulk of those overlapped with colitis-responsive genes. In contrast, consumption of TWD led to a prolonged transcriptional response of immune-related genes, with 305 DEGs identified at recovery compared to pre-DSS. Of note, relatively few DEGs were identified for mice fed either diet when comparing recovery to the colitis time point, although a comparison of TWD-fed to AIN-fed mice at recovery revealed 48 genes that were uniquely expressed in response to TWD (Figure 6C.iii).

**Figure 7:**
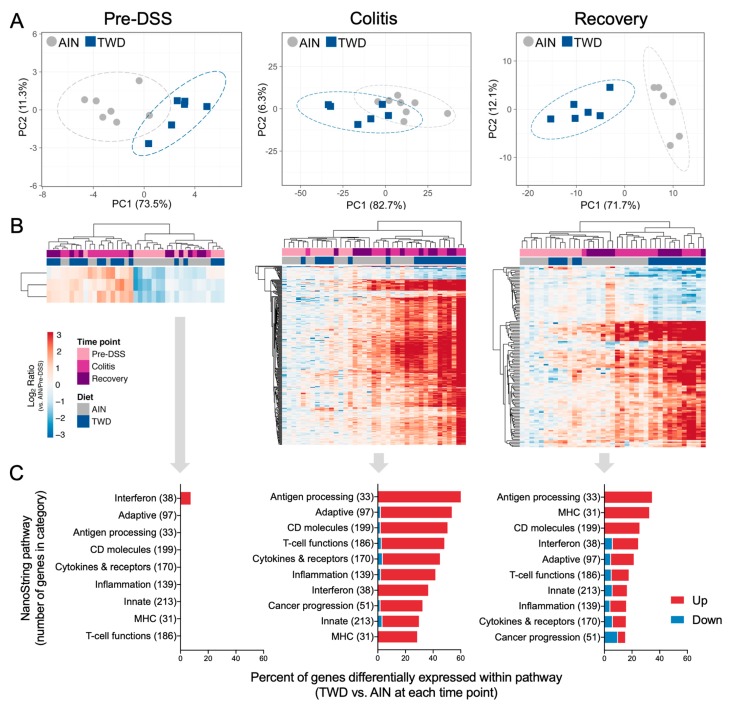
Exposure to the TWD prior to, during and after DSS-induced gut injury intensified changes in expression of genes associated with immune and cancer pathways as compared to mice fed AIN (Experiment E). (**A**) Principal components analyses (PCA) of differentially expressed genes (BH *q* < 0.05, log_2_ ratio > 1 or < −1) in colon tissues of mice fed either AIN or TWD prior to DSS (Pre-DSS), during active colitis (Colitis), or two weeks later during recovery (Recovery). PC1 and PC2 are shown with the 95% prediction ellipse. Because only three genes met all significance criteria at the Pre-DSS time point, all genes differently expressed by at least two-fold with an FDR *p* < 0.10 were used for the PCA analysis for that time point only. (**B**) Unsupervised, bidirectional hierarchical cluster analysis of colon transcriptome data using the Euclidean distance method with average linkage. For each time point, the heatmap shows all genes differentially expressed between TWD and AIN diet groups colored by the log_2_ ratio calculated with respect to AIN/Pre-DSS. (**C**) Bar charts indicate the number of genes differentially expressed at each time point for selected immune and cancer pathways. The total number of genes affiliated with each pathway for the PanCancer Immune panel are indicated in parentheses.

Principal components analyses of DEGs at each time point indicated that the transcriptional responses in colon mucosa of TWD-exposed mice were moderately distinct from those fed AIN at colitis, but were highly divergent at the recovery time point (Figure 7A). Similar results were obtained by unsupervised, bi-directional hierarchical clustering of DEGs at each time point (Figure 7B), with noticeable segregation of expression profiles by diet group at colitis and marked separation at recovery. Figure S7 shows both PCA and hierarchical clustering for all DEGs across all time points as single plots, revealing distinct patterns of immune-related gene expression for pre-DSS and other time points and some separation of TWD- and AIN-fed mice at colitis and recovery.

**Figure 8:**
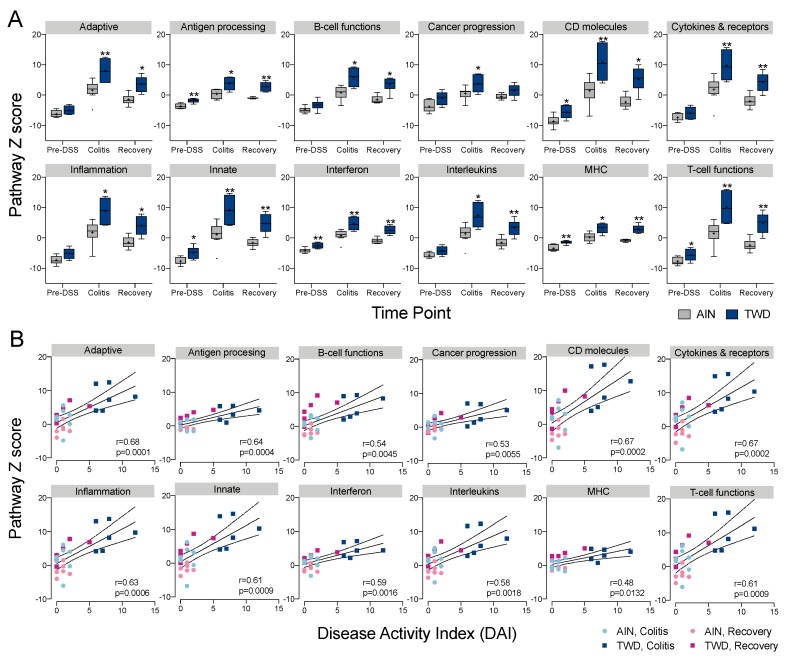
Pathway Z scores indicate broad scale changes in transcript abundance for genes associated multiple immune and cancer pathways that are also highly correlated with the colitis DAI. (**A**) Colon tissue gene expression pathway Z scores at Pre-DSS, colitis and recovery time points for mice fed AIN or TWD diets. Scores are shown as Tukey box plots (*n* = 6 to 8) for selected immune and cancer pathways. * *p* < 0.05 and ** *p* < 0.01 compared to time point-matched AIN control as determined by the non-parametric Steel-Dwass test. (**B**) Correlation analyses of immune-associated pathways and colitis disease activity index (DAI) at colitis and recovery time points. Spearman correlation *r* and *p* values are indicated.

Only a few DEGs were identified in mice fed TWD prior to DSS treatment, including *Ifit2*, *Ifit* 3 and *Nlrc5*, all three of which are involved in interferon response (Figure 7C). At colitis and recovery, DEGs were associated with antigen processing, adaptive immune responses, innate immune responses, T-cell function, CD molecules, interferon and MHC pathways (Figure 6C). Expression heatmaps depicting DEGs for these pathways are provided in Figures S8 and S9. Global and directed pathway significance scores showed that responses to TWD were markedly greater for both colitis vs. pre-DSS and recovery vs. pre-DSS comparisons with most pathways activated by TWD exposure (Figure S10). Similar results were obtained when pathway-specific transcript abundance was analyzed as the Z-scaled first principal component (the pathway Z score). Affected genes were associated with multiple immune and cancer pathways, including CD molecules, T-cell functions, cytokines and receptors, and adaptive and innate immunity (Figure 7A). Of note, several pathways appeared differentially regulated by TWD at the pre-DSS time point, including antigen processing, CD molecules, interferon, MHC and T-cell functions, suggesting that the pathway response overall was divergent from AIN, even though few DEGs were identified using the selected FDR and fold change criteria. Pathway Z scores were strongly correlated with the DAI index, most notably for adaptive, CD molecules, cytokines and receptors, inflammation, innate and T-cell functions pathway (Figure 8B).

**Figure 9:**
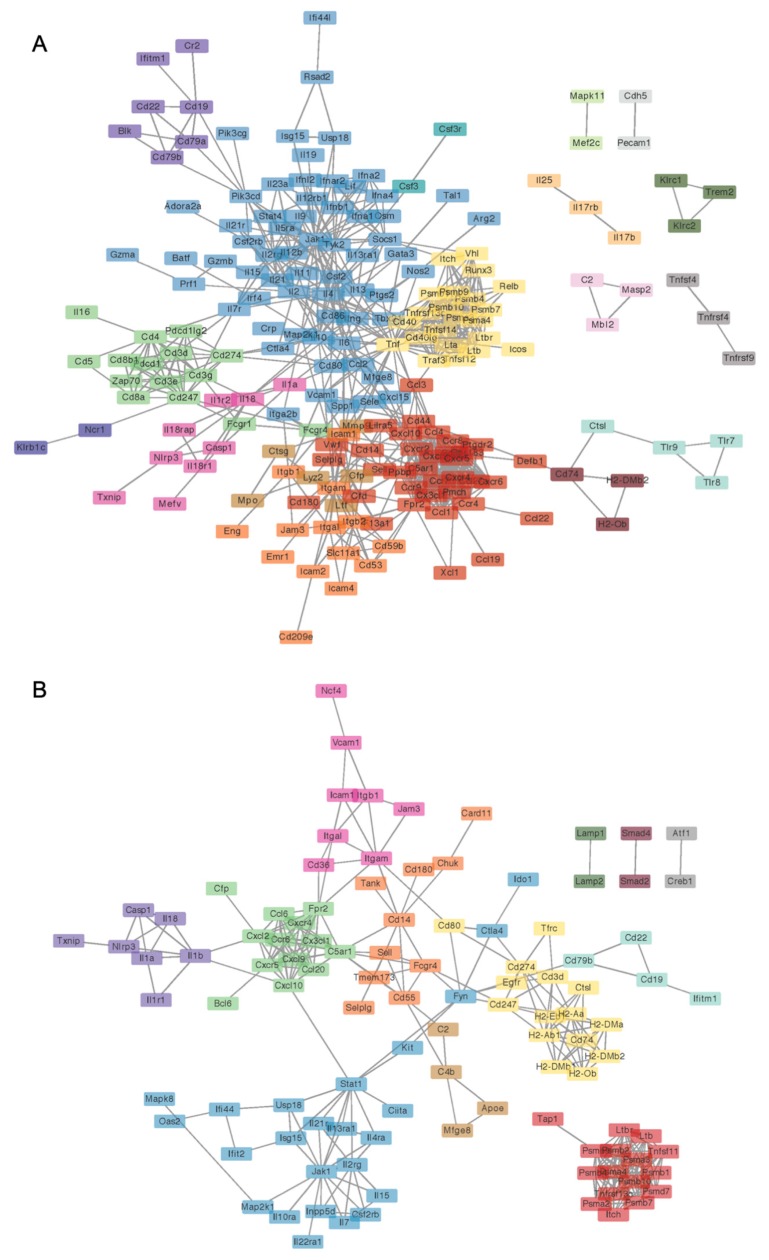
(prior page) Network visualization of significant differentially expressed immune response genes in colon tissues of mice fed TWD as compared to their AIN counterparts at colitis (**A**) or recovery (**B**). Network of DEGs in mice fed TWD Network interactions were modeled using the STRING database (string-db.org) with a minimum required interaction score ≥0.9, and clusters were identified using the Markov Cluster (MCL) algorithm with inflation parameter of 1.5. The network was visualized in Cytoscape. Major groupings include transcripts associated with cellular response to cytokine-mediated cell signaling and the STAT cascade (blue), antigen processing (yellow), cellular protein catabolic processes (red), regulation of granulocyte chemotaxis (green and purple).

Because the NanoString annotated pathways are broadly descriptive of immune-related functions, we explored gene ontology enrichment analysis, which provides greater resolution and more specific insight into biological processes affected by DEGs. Circos plots generated using lists of DEGs for AIN or TWD at the colitis timepoint also reveal substantial overlap in transcriptional profile for these two diets, with a large majority of DEGs for the AIN diet also regulated in mice fed TWD (Figure S11). However, a large fraction of DEGs for mice fed TWD were unique, with relatively few overlapping GO biological process terms with the AIN diet group (Figure S11). Alternatively, at the recovery time point, while many more genes were uniquely expressed in TWD-fed mice, a greater fraction of those unique transcripts were associated with similar GO biological process terms. For mice fed AIN, notable biological processes that were significantly enriched at colitis in mice fed the AIN diet included inflammatory response (GO:0006954), cell chemotaxis (GO:0060326), second messenger-mediated signaling (GO:0019932), vascular endothelial growth factor production (GO:0010573), and leukocyte migration involved in inflammatory response (GO:0002523), among others (Figure S11). Enrichment of some of these processes persisted to the recovery time point, although some new terms were identified as well, such as interferon-gamma production (GO:0032609) and defense response to bacterium (GO:042742). Biological process terms corresponding to the subset of genes similarly regulated at colitis in both AIN and TWD-fed mice were consistent, with cell chemotaxis the top term for TWD-exposed mice at both time points. Although some terms appear unique for TWD, one should note that the terms shown represent a cluster defined by semantic similarity with very similar genes (e.g., calcium-mediated signaling (GO:0019722) and second messenger-mediated signaling (GO:0019932)) (File S6). However, the TWD DEGs were associated with some biological processes that appeared exclusive to that diet group, such as T cell activation (GO:0042110) and T cell differentiation involved in immune response (GO:0002292) at the colitis time point or T-helper 17 type immune response (GO:0072538) and defense response to protozoan (GO:0042832) at the recovery time point. To gain further insight into processes associated with the transcriptional response unique to TWD exposure, ontology analyses were also performed using only the set of DEGs that were not also regulated in AIN-fed mice at both colitis and recovery time points (Figure S12). During colitis, the few overlapping GO terms are likely connected with second messenger signaling, as suggested by the high significant enrichment for calcium-mediated signaling (GO:0019722). Genes with non-overlapping GO terms were associated with processes including T cell activation (GO:004211) and T cell cytokine production (GO:0002369). At recovery, although substantially more overlap in biological process terms for the different diet gene sets was evident, the DEGs unique to TWD were associated most strongly with B cell activation (GO:0042113) and T-helper 17 type immune response (GO:0072538)—terms that did not appear in any of the AIN ontology results (File S6). These trends were largely confirmed by STRING-db network mapping, which showed that genes associated with cytokine-mediated cell signaling, B-cell activation and lymphocyte proliferation, leukocyte migration involved in inflammatory response, and regulation of granulocyte chemotaxis were most predominantly impacted by exposure to TWD at the colitis time point (Figure 9A). At recovery, DEGs involved in cytokine-mediated cell signaling, antigen processing, cellular catabolic processes, and regulation of granulocyte chemotaxis and regulation of T-helper 2 cell differentiation were identified (Figure 9B). Network maps for colitis vs. pre-DSS and recovery vs. pre-DSS time points within each diet group are provided in the supplementary material (Figures S11–S14). Additionally, visualization of DEGs mapped to top KEGG pathways, including inflammatory bowel disease (mmu05321), cytokine-cytokine receptor interaction (mmu04060), the NF-Kappa B signaling pathway (mmu04151), and the cell adhesion pathway (mmu04514) are provided for each diet for colitis vs. pre-DSS or recovery vs. pre-DSS time point comparisons (Figures S15–S22).

**Figure 10:**
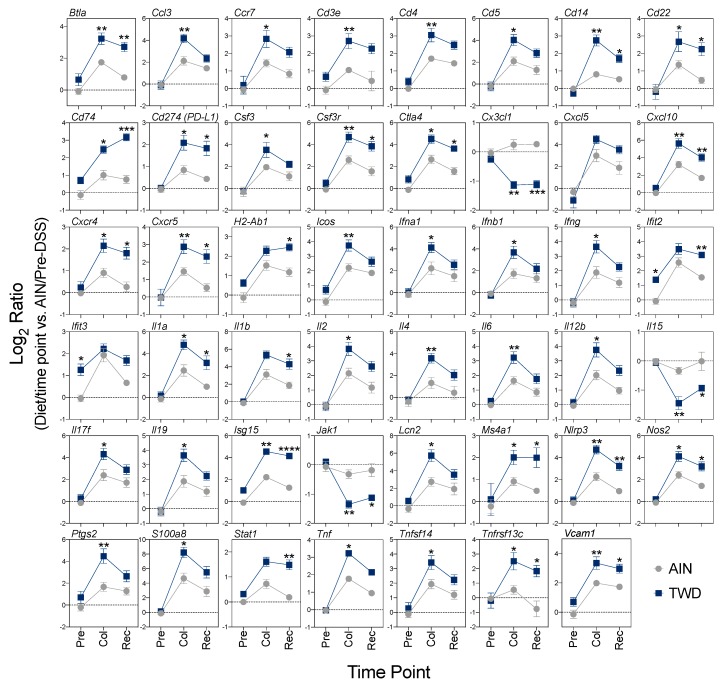
Expression profiles for selected genes of interest associated with immune and inflammatory responses. Values shown are the average log_2_ ratio ± SEM for selected genes calculated with AIN/Pre-DSS as the reference. Significant differences in expression (FDR-corrected *q*-value < 0.05 and log_2_ ratio > 1 or < −1) between TWD and AIN diets at each time point are noted as follows: * *q* < 0.05; ** *p* < 0.01; *** *p* < 0.001; **** *p* < 0.0001. Statistical results for comparisons across time points within each diet group are not shown here, but are available in File S2 along with the specific FDR *q*-values for the significant observations noted in the figure. Pre, pre-DSS; Col, colitis; Rec; recovery.

Comparisons of the impact of AIN and TWD on representative genes over the course of colitis onset and recovery are depicted in Figure 10. An examination of expression for selected genes representative of these pathways reveals two patterns of interest. First, responses of these DEGs were routinely more robust in mice fed TWD than AIN during colitis, as noted for genes associated with interferon response (*Ifna1*, *Ifnb1*, *Ifng*, *Nos2*), inflammation (*Il1a*, *Il4*, *Il6*, *Il17f*, *Il12b*, *TNF*, *Nlrp3*, *S100a8*), and chemokines and their receptors (*Ccl3*, *Ccr7*, *Cxcl10*, *Cxcr4*, *Csf3*, *Csf3r*), among others. Second, many genes differentially expressed between TWD and AIN diets at colitis were still upregulated at the recovery time point compared to pre-DSS, though not markedly more so in TWD-fed mice (e.g., *Ifna1*, *Il6*, *Tnf*). However, a number of transcripts were found to be uniquely over- or under-expressed in TWD-fed mice at recovery compared to their AIN counterparts, including several DEGs associated with antigen processing and T-cell function pathways (*Cd5*, *Vcam1*, *Cd247*, *Cd74*, *Btla, H2-Ab1*), interferon (*Ifit2*, *Isg15*, *Nos2*), inflammation (*Il1a*, *Il1b*, *Il3ra1*, *Il15*, and *Nlrp3*), and B-cell activation (*Cxcr5*, *Cd22*, *Ms4a1*, *Tnfsf13b*). Prolonged activation of these pathways through recovery from DSS-induced colitis correlated well with observed disease activity (Figure 8B).

**Figure 11:**
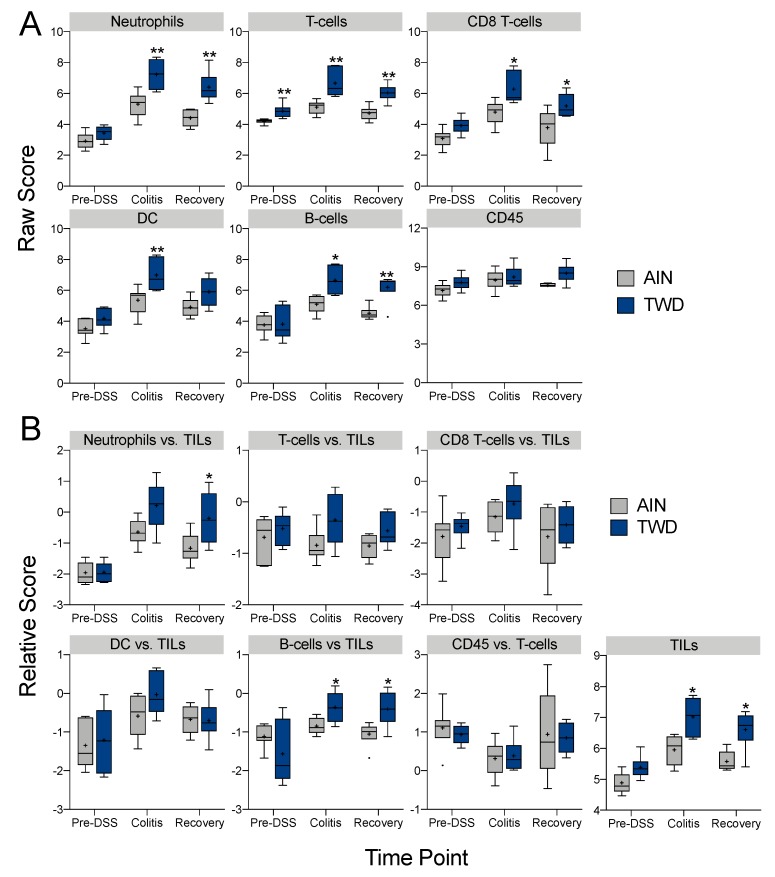
Profiling of immune cell types in colon tissues obtained at pre-DSS, colitis or recovery time points from mice fed either AIN or TWD. (**A**) Data shown are the log_2_ scores for the indicated cell types, and only those cell types that met quality control testing for correlation of marker gene expression (*p* < 0.05) are shown. Scores may be compared within a cell type for both diet and time point, but comparisons across cell types are not appropriate for this method of quantitation. Scores do not convey information about the absolute number of cells in the samples. (**B**) Data shown are the relative cell type measurements with respect to total infiltrating leukocytes (TILs). * *p* < 0.05 and ** *p* < 0.01 compared to time point-matched AIN control as determined by the non-parametric Steel-Dwass test.

Immune cell type profiling in colon mucosa based on the expression of cell-specific transcripts was performed using NanoString software (Figure 11). Consistent with our prior histopathological assessment of inflammation in colon tissues of mice provided DSS (Figure 1J and Figure 5), neutrophils increased in both AIN- and TWD-fed mice during colitis, though significantly more so in mice provided TWD (Figure 11A). Furthermore, in agreement with our prior observations, this apparent increase in neutrophil abundance in TWD-fed mice persisted through recovery from DSS-induced inflammation and gut injury. Similar patterns were evident for other immune cell types, including T-cells, CD8 T-cells, and B-cells, though not apparently for all CD45+ cells. When considered as a fraction of the total population of infiltrating leukocytes (TILs) in the tissue, B-cells were enriched in TWD-fed mice at colitis while both B-cells and neutrophils were enriched at recovery (Figure 11B).

## 4. Discussion

Herein, we report that a rodent diet that recapitulates U.S. nutrition patterns with respect to macro- and micronutrient content markedly enhanced colon tumorigenesis in multiple mouse models of CAC as compared to a basal diet optimized for rodent health. This study is the second from our group to show that the TWD functions to promote colon cancer in mice. Previously, we showed that A/J mice initiated with AOM and fed TWD for 16 weeks developed significantly more aberrant crypt foci and total crypt cells than mice fed the AIN93G diet [24]. Now, we report that consumption of TWD significantly enhanced tumorigenesis in C57BL/6J mice using both the AOM + DSS and *Apc^Min/+^* + DSS models of CAC, whereas small intestinal tumorigenesis was not affected. Furthermore, this increased tumor response was attributed to the micronutrient fraction of the TWD, and restoration of calcium and vitamin D to standard amounts ameliorated the tumor-promoting effects of the TWD. Exposure to the TWD markedly enhanced DSS-induced symptoms of colitis, exacerbated inflammation and mucosal injury pathologies in colon tissues, and delayed tissue repair after cessation of DSS exposure. This study is the first to assess the dynamics of immune and cancer pathway gene expression during the onset and resolution of DSS-induced colitis and the first to employ highly multiplexed, direct digital detection NanoString technology for analysis of a colitis transcriptome in mouse colon mucosa. Results of these targeted gene expression analyses point to striking up-regulation of hundreds of genes associated with interferon response, inflammation, innate immunity, adaptive immunity, and chemokines and receptor pathways in mice fed TWD as compared to the standard AIN diet during active colitis. In a pattern that mirrored the persistent elevation in inflammation and mucosal injury observed in the AOM + DSS longitudinal study, dysregulation of many of these genes persisted through recovery from gut injury, in addition to the stimulation of other pathways, such as B-cell activation and antigen processing. Collectively, these data suggest that enhanced and sustained inflammation and dysplasia of the colon mucosa is a probable mechanism for promotion of CAC by the TWD.

As we reported previously, mice consuming the DIO diet acquired an obesity/metabolic syndrome phenotype whereas consumption of the TWD did not significantly alter any biomarkers of metabolic health [36], a somewhat surprising observation considering that the TWD diet contains substantially more fat than the AIN93G diet (35% compared to 17% of total kcal, respectively). Thus, the colon tumor-promoting effect of the TWD was unrelated to a metabolic syndrome phenotype or systemic inflammation induced by consumption of a high fat diet. Furthermore, our observations also showed that the high fat DIO diet did not enhance colon cancer in the AOM + DSS mouse model, a finding somewhat contrary to prior observations for development of preneoplastic lesions in rats [37,38]. The results of these comparisons of the high fat DIO diet to the TWD point to the critical need to employ a diet model that more broadly represents typical human nutrition. Had the first experiment design included only the DIO diet as the model Western diet, we could have easily (and erroneously) concluded that colorectal cancer risk in C57BL/6J mice was unaffected by the Western nutrition pattern. Moreover, we would have missed the noteworthy discovery that inappropriate micronutrient consumption strongly increased colon tumorigenesis in this mouse model, as evidenced by highly similar results for the TWD and the VMM diets, both of which had vitamin and mineral content that reflects typical American nutrition on an energy density basis. Modification of macronutrient content alone (i.e., DIO and MM diets) did not increase colorectal tumorigenesis, an observation that also points to a critical role for micronutrients in promoting colorectal carcinogenesis in this pre-clinical mouse model.

The nutrient density approach was previously employed by Newmark et al. [18] to construct a Western-type diet containing the amounts of fat, phosphorous, calcium, and vitamin D observed in typical American diets. Newmark’s Western diet promoted spontaneous hyper-proliferation and hyperplasia in colonic epithelium of aged C57BL/6J mice. In a subsequent study, this group further modified this experimental diet by reducing amounts of nutrient components critical for methyl donor generation, including folic acid, methionine, and vitamin B_12_ [20]. Consumption of this modified western diet led to higher rates of spontaneous colon adenoma and carcinoma development in normal mouse colon. In formulating the TWD, we expanded upon this nutrient density approach for diet formulation to include the complete array of macro- and micronutrients included in commercial semi-purified rodent diets [23]. On an energy density basis, amounts of vitamins B_6_, B_12_, and folate in the TWD are about 33% to 50% lower as compared to the AIN93G control diet, while calcium and vitamin D levels are approximately 60% lower. In experiment B, we sought to determine if certain micronutrients could be added back to the TWD to block the tumor-promoting phenotype, following an approach somewhat like that of Newmark’s group [18]. We hypothesized that TWD-enhanced tumorigenesis could be abrogated by adding back to the TWD the original amounts of calcium and vitamin D or dietary methyl donors (B_2_, B_6_, B_12_, folate and choline) to match the AIN93G diet. The addition of calcium and vitamin D to the TWD decreased colitis disease activity, tumor multiplicity and burden compared to mice fed the unmodified TWD. The addition of methyl donor micronutrients to the TWD resulted in a modest reduction in colitis and tumorigenesis, but there were no significant differences in any of the study endpoints between mice fed the methyl donor-supplemented TWD and the unmodified TWD (Figure 2A–C). Because the TWD models the typical U.S. diet with respect to nearly all major micro- and macronutrient components, it is not possible to conclude that these several micronutrients we investigated here are exclusively responsible for the tumor-enhancing effect of TWD. Nevertheless, our strategy is an important advance on prior studies that investigated the impact of only one or a select few components of the Western diet on colon cancer.

As noted above, evidence from human epidemiological studies and experiments in animal models points to a role for calcium and vitamin D in modulation of CRC [39,40,41]. A review of epidemiological evidence suggests that dietary intake of not more than 1000 mg/day calcium and 1000 to 2000 IU/day vitamin D may be protective against CRC [42]. In 2006, Wactawski-Wende reported that results from the Women’s Health Initiative, the largest randomized controlled clinical intervention to date, suggested that combined intake of 1000 mg/day calcium and 400 IU/day vitamin D_3_ over seven years did not reduce CRC incidence [43]. However, reanalysis of these data accounting for the personal use of supplements revealed that the calcium and vitamin D intervention indeed improved total cancer outcome, including CRC incidence [44].

Calcium functions to regulate cellular signaling, cell proliferation and cell growth, while vitamin D is critical for adequate uptake of calcium in the small intestine. A 24-week study using *Apc^1638N^*^/+^ mice, which have a truncation on codon 1638 of the tumor suppressor *Apc* gene, found that feeding a modified AIN76A diet with half the content of vitamin D and 90% less calcium resulted in the formation of colonic adenomas and carcinomas and enhanced expression of cyclin D1 and anti-apoptotic protein Bcl-2, which are commonly overexpressed in colorectal cancer [45]. In a different study using this same diet fed to C57BL/6J mice for 3 or 6 months, researchers identified important transcriptome changes associated with induction of the oxidative stress response pathway [46]. Moreover, calcium and vitamin D were determined to be important regulators of bile acid synthesis and excretion. Vitamin D has been shown to be involved in the detoxification of bile acids through the regulation of fibroblast growth factor 15 [47] and the vitamin D receptor [48] in the intestine, while calcium has been shown to increase fecal fatty acid excretion [49,50]. Bile acids contribute to colorectal carcinogenesis by increasing cellular oxidative stress, and they have been associated with the promotion of cell populations resistant to their apoptotic effects [51]. Bile acids are the main end products of cholesterol catabolism in mammals, and, hence, they are generally found in high levels in individuals that consume a high-fat diet [52]. Thus, the combined impact of excess fat with low calcium and vitamin D content in the TWD could explain the promoting effect of this diet on colon tumorigenesis observed in this study.

Numerous studies have linked vitamin D deficiency with increased risk of IBD, a major risk factor for development of CRC [53,54,55,56,57], although it is unclear whether this deficiency is a cause of IBD or a consequence. However, there is ample evidence supporting a role for the bioactive form of vitamin D, 1,25-dihydroxycholecalciferol (D_3_), as a potent immune and inflammation modulator in the gastrointestinal tract. D_3_ has been shown to have an important role in maintaining gut homeostasis by reducing inflammation, including reduction in the activation of T_h_1 and T_h_17 cells and suppressing activation of pro-inflammatory cytokines IL-1, IL-6, IL-8, and TNF [reviewed in 57–59]. Vitamin D deficiency shifts this system toward a pro-inflammatory state that favors T_h_1 and T_h_17 activation and increased expression of TNF, IFNγ, and IL-17, as well as elevated expression of pro-inflammatory Il-4, IL-5 and IL-13, and gut barrier dysfunction. In this study, the pattern of gene expression in TWD-fed mice (vitamin D depleted to just 40% of the AIN diet), generally fits well with this pro-inflammatory model of vitamin D deficiency. During colitis, TWD increased expression of *Tnf* by 2.5-fold, *Il1b* by 2.8-fold, *Il4* by 4.8-fold, *Il6* by 3.3-fold, *Ifng* by 3.8, and *Il17f* by 4.2-fold as compared to mice fed the AIN diet. Furthermore, gene ontology analyses revealed activation of the T-helper 17 type immune response during recovery from DSS-triggered gut injury in mice fed TWD, but not those provided AIN. Although mucosa samples for experiment B were not available for gene expression analyses, we can surmise that the restoration of vitamin D (along with calcium) in this experiment likely reversed the pro-inflammatory effects of the vitamin D deficient TWD diet on mucosal gene expression and allowed for a shift towards gut homeostasis, as suggested by the observed decrease in symptoms of colitis following DSS treatment.

Choline, folate, and vitamins B_6_ and B_12_ are essential in cellular biosynthetic pathways due to their roles as donors of methyl groups for one carbon-metabolism [58], and consequently influence DNA methylation, cycling of *s*-adenosylmethionine, activity of DNA methyltransferases and miRNAs with both oncogenic and tumor suppressive functions [59]. These methyl donor micronutrients are present in the AIN93G diet at substantially greater levels than the RDA value for humans, when compared on an energy density basis [23]. Moreover, the TWD contains substantially lower levels of these micronutrients than are present in the AIN93G diet. A case-control study of subjects from the Multiethnic Cohort study found that people in the highest quartile for pyridoxal-5′-phosphate intake (active form of B_6_) had a 48% reduction in CRC risk [60]. A meta-analysis that included 7 cohort-studies and 9 case-control studies found a 25% lower risk of CRC among those in the highest category of dietary folate intake compared with those in the lowest category, while only a 5% lower risk was observed for total folate intake (including supplements) [61]. An animal study using male Sprague-Dawley rats fed with either a diet with 50 μg/kg diet of vitamin B_12_ or a diet with no vitamin B_12_ found that, after 10 weeks of feeding, rats fed the deficient diet displayed a 35% decrease in genomic methylation and a 105% increase in base substitution of uracil. Although some studies have found weak or no association between CRC risk or development and dietary B-vitamins [62,63,64], the supporting evidence points to a complex interaction between other nutrients in the diet. Furthermore, the low levels of B-vitamins in the TWD could explain the promoting effect on tumorigenesis observed in animals fed this diet.

To determine whether increased CRC associated with the TWD was specific to the AOM + DSS model, we fed *Apc^Min/+^* either AIN, DIO, or the TWD. *Apc^Min/+^* mice are a genetic model of CRC that develop intestinal tumors spontaneously without administration of AOM. Similar to our previous investigations, the TWD increased colon tumor multiplicity and burden relative to both mice fed either the AIN or DIO diets. However, there were no differences between diet groups for small intestinal tumorigenesis. These results confirm our earlier findings and suggest CRC phenotype associated with the TWD is not due to interactions between dietary factors and metabolism of the AOM carcinogen.

The histological assessment of colon tissues revealed that consumption of the TWD enhanced inflammation over the progression of colitis, through recovery to tumorigenesis. Thus, we sought to gain insights into the underlying mechanisms for TWD-enhanced tumorigenesis via targeted multiplex gene expression analysis using the nCounter transcript counting platform. For mice fed the standard AIN diet, results of these analyses confirmed that DSS-induced colitis was associated with broad changes in expression of genes associated with adaptive, innate, inflammation, interferon, and chemokines and receptors pathways, with many of these responses persisting, albeit to a lesser extent, through the recovery phase 14 days after cessation of DSS treatment. Remarkably, exposure to the TWD exacerbated these responses across the board such that many more DEGs were identified at colitis and recovery stages with markedly greater changes in expression as compared to the pre-DSS controls. When considering the overall profile of gene expression, segregation of the diet groups was evident, even prior to the induction of colitis by DSS. The apparent distinct gene expression profiles following three weeks of feeding TWD were likely caused by the upregulation of several genes involved in interferon response (*Ifit2*, *Ifit3*, and *Nlrc5*), as well as other more modest, but not significant, changes in genes associated with complement, leukocyte function and MHC pathways suggested by global significance scores. Exposure to the TWD may prime colon tissue toward a more responsive state such that the immune and inflammation responses upon challenge with DSS were exacerbated compared to mice fed the standard AIN diet. Furthermore, very distinct transcriptional responses were evident during recovery from DSS-induced gut injury, most obviously revealed by principal components analysis, which supports prior histological observations that TWD exposure impeded the repair of colon mucosa and reduction in inflammation. Moreover, cell type profiling suggested that colon mucosa of TWD-exposed mice was typified by an increase in total infiltrating leukocytes, including elevated neutrophils, T-cells, dendritic cells, and B-cells, with an apparent enrichment in the relative abundance of B-cells during colitis and recovery. Interestingly, Bergomas, et al. [65] reported that chronic inflammation induced in the AOM + DSS mouse model of CAC was associated with an increase in amount of lymphoid tissue in the colon mucosa and the development of intratumor tertiary lymphoid structures with highly compartmentalized B-cells, T-cells, mature dendritic cells and a network of CD21^+^ follicular dendritic cells.

Given that the PanCancer Immune Profiling probe set captures expression data for up to 750 genes, we sought to compare the transcriptional profiles for our TWD-exposed mice during colitis to other available datasets in animal models and human UC patients. Our comparison focused primarily on those studies using genome-wide methodologies including a genome-wide microarray study of mouse colon mucosa collected after 6 days exposure to 3% DSS [66], RNAseq analysis of colon mucosa collected after exposure to 4% DSS (5 days treatment, with collection on day 8) [67], RNAseq analysis of colon mucosa from the PAC-*Il10^-/-^* mouse model compared to wildtype [67], a microarray study of colon tissue samples from human UC patients compared to healthy controls [68], and an RNAseq assessment of colon tissues from human UC patients versus healthy controls [67]. (We note that other reports also employed genome-wide analyses to examine the colon transcriptome in DSS or IL-deficient mice, although the complete results or underlying data were not publicly available for us to use in this comparative analysis). When comparing the transcript profiles across these various studies, a list of 19 commonly regulated genes were identified including *C3*, *Ccl2*, *Ccl3*, *Ccl4*, *Ccr2*, *Cxcl1*, *Cxcl10*, *Cxcl2*, *Cxcl5*, *Cxcl9*, *Icam1*, *Il1b*, *Lcn2*, *Mmp9*, *Nos2*, *S100a8*, *Stat1*, *Tnf*, *Vcam1* (Table S1).

**Figure 12:**
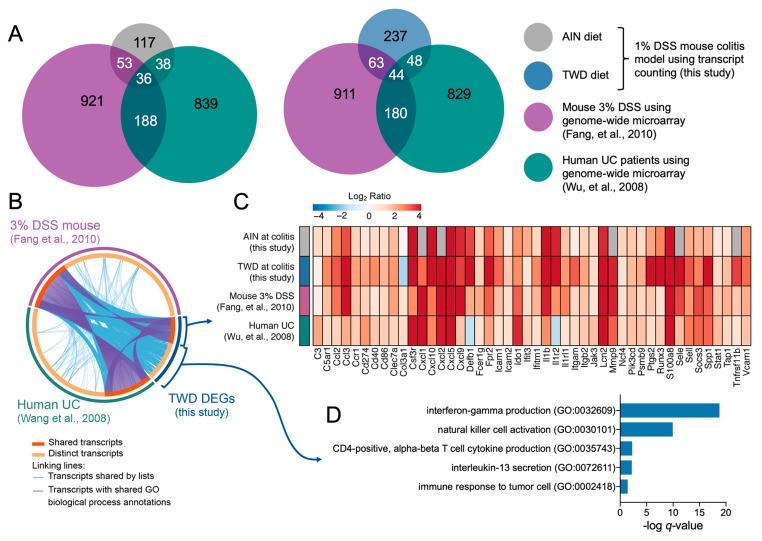
Identification of common colitis-responsive transcripts in dextran sodium sulfate (DSS) models of colitis and human ulcerative colitis (UC) patients. Expression data from this study were compared to prior reports using genome-wide microarray platforms as noted in the Materials and Methods. (**A**) Venn diagrams depicting overlap in differentially expressed genes (DEGs) are shown comparing colitis-responsive transcripts in mice fed the AIN93G (AIN) diet or total Western diet (TWD) to DEGs identified using a 3% DSS model of colitis in C57BL/6J mice (purple) [68] or DEGs identified in colon mucosa of human UC patients [70]. (**B**) Circos plot showing overlap of DEGs (purples lines) and overlap of shared ontology terms (blue lines) for TWD DEGs at colitis compared to DEGs for the 3% DSS mouse model or DEGs identified in human UC patients. (**C**) Heat map depicting the log_2_ fold change values for those DEGs identified as common to each transcriptional profile. Note that the AIN and TWD DEG lists were merged to generate the heatmap, resulting in a total of 46 genes. Gray boxes within the AIN data set indicate genes that did not meet the detection threshold for nSolver data analysis. (**D**) Gene ontology biological process terms significantly enriched (*p* < 0.05, enrichment minimum 2) in list of 237 DEGs unique to TWD at colitis (this study) as determined using the complete probe set as the background gene list (Metascape).

Because the mouse DSS colitis model most closely approximates human UC, we compared the transcript profile obtained in this study using the NanoString platform to the list of DEGs identified by Fang, et al. [66] using the 3% DSS model and to DEGs identified by Wu, et al. [68] in human UC patients (Figure 12). A larger set of commonly regulated genes was discovered (46 total), with notable consistent expression for many transcripts across all studies, including *Csf3r*, *Cxcl2*, *Cxcl5*, *Ido1*, *Il1b*, *Lcn2*, *Mmp9*, *S100a8*, and *Spp1* (Figure 11B,C). Alternatively, a large portion of the colitis TWD-regulated gene set was unique to this study, as these genes did not meet significance criteria in either the mouse DSS or human UC genome-wide studies (Figure 12B,D). Interestingly, these unique DEGs are annotated with GO biological process terms associated with IFNγ regulation and secretion (GO:0032609), IL-13 secretion (GO:0030101), and immune response to tumor cell (GO:0002418) (Figure 12D). However, we did note very high overlap in biological processes for all the DEGs identified at colitis in TWD-fed mice (note blue lines in Figure 12B), suggesting functional redundancy captured by other DEGs in the comparison studies. The differences in DEGs identified in our study compared to others using DSS to induce colitis may be related to the amount and duration of DSS treatment (1% for 10 days in this study versus 3% to 4% for 5 to 8 days in other reports). Or, the differences may be due to the nature of the NanoString technology, which allows for detection of small populations of mRNA, lacks bias that can be introduced through sample processing and amplification, and has the capacity for detecting transcripts in less-than-ideal quality samples.

This work has some limitations. First, our animal studies focused on the DSS-induced model of colitis-associated colorectal cancer, and we have not yet explored the impact of TWD in the *Il10^-/-^* mouse model of spontaneous colonic inflammation. Results of the comparative transcriptomics analyses across DSS and IL-10 deficient mouse models, however, suggest that our TWD-enhanced DSS colitis model shares some similar transcriptional responses as observed in *Il10^-/-^* mice. Furthermore, we have investigated the effects of TWD in two mouse strains to date (C57BL/6J in this study and A/J males in [24]). However, given the influence of genetic background on susceptibility to inflammation and the progression of disease [69,70], a more extensive evaluation of strain-dependent responses to the TWD in context of gut inflammation would be valuable for future work. Our transcriptomics approach leveraged a reasonable sample size and a limited gene target set for a relatively high-powered analysis of mucosal gene expression in this pre-clinical animal model of CAC. The NanoString PanCancer Immune Profiling probe set was selected based on the hypothesis, supported by histopathological evidence, that TWD exposure enhanced colonic inflammation. On the other hand, this targeted transcriptomics approach limited our possible observations to genes related to immune response or inflammation processes. There remains the possibility that other unrelated pathways were affected by exposure to the TWD during colitis or recovery from gut injury. Last, the experiments included herein did not include an assessment of the microbiome composition nor manipulations of the gut microbiome (e.g., antibiotic exposure) to determine the contribution of gut bacteria to TWD-driven changes in gut inflammation. While of high interest to our group, these additional experimental factors and analyses were beyond the scope of the present work.

## 5. Conclusions

Taken together, our observations indicate that consumption of the TWD markedly enhanced colitis, delayed recovery from gut injury, and promoted colon tumorigenesis. Furthermore, these effects were associated with extensive changes in expression of immune-related genes in the colon mucosa. The use of a basal diet that more completely modeled Western nutrition on an energy density basis revealed that the micronutrient fraction of the diet—primarily vitamin D and calcium, and, to a lesser extent, methyl donor micronutrients—was the driving force in promoting gut inflammation. Evidence from this work supports the idea that a rodent diet more representative of the diet consumed by the majority of Americans is necessary to appropriately evaluate colon cancer risk and to develop specific and effective prevention strategies [71]. Results of studies using the TWD may result in pre-clinical findings that can be more accurately translated to at-risk populations. Moreover, future work by our team will focus on the contribution of the TWD to the composition of the gut microbiome in context of colitis onset, recovery from gut injury, and subsequent tumorigenesis, as well as the potential benefit of various dietary interventions with functional foods to modulate the gut microbiome and suppress Western-diet enhanced CAC. Furthermore, given the profound impact of the TWD on colon carcinogenesis and the mounting evidence for perinatal or transgenerational impacts of diet on cancer risk, we are also investigating the impact of TWD provided via the maternal diet on risk of cancer to offspring.

## Figures and Tables

**Table 1:** Composition of experimental diets.

	AIN93G (AIN)	TWD	DIO	VMM	MM	TWD + CaVD	TWD + MD
Energy density (kcal/g)	3.8	4.4	4.6	3.7	4.4	4.4	4.4
Macronutrients							
*Carbohydrates (g/kg diet)*							
Corn starch	398	230	85	398	230	230	230
Maltodextrin	132	70	115	132	70	70	70
Sucrose	100	261	200	100	261	261	261
Cellulose	50	30	58	50	30	30	30
kcal (% of total)	60.1%	54.5%	36.2%	60.1%	54.5%	54.5%	54.5%
*Proteins (g/kg)*							
Casein	200	190	245	200	190	190	190
L-cystine	3	2.8	3.5	3	2.8	2.8	2.8
kcal (% of total)	18.8%	15.4%	19.0%	18.8%	15.4%	15.4%	15.4%
*Fats (g/kg)*							
Soybean oil	70	31.4	30	70	31.4	31.4	31.4
Anhydrous milk fat	0	36.3	0	0	36.3	36.3	36.3
Olive oil	0	28.0	0	0	28.0	28.0	28.0
Lard	0	28.0	195	0	28.0	28.0	28.0
Beef tallow	0	24.8	0	0	24.8	24.8	24.8
Corn oil	0	16.5	0	0	16.5	16.5	16.5
Cholesterol	0	0.4	0	0	0.4	0.4	0.4
kcal (% of total)	17.2%	34.5%	44.8%	17.2%	34.5%	34.5%	34.5%
Micronutrients							
*Minerals (mg/kg)*							
Calcium	5000	2011	5000	2011	5000	5000	2011
Phosphorus	3000	2757	3000	2757	3000	2757	2757
Sodium	1019	7078	1019	7078	1019	7078	7078
Potassium	3600	5333	3600	5333	3600	5333	5333
Magnesium	507	589	507	589	507	589	589
Iron	35	31	35	31	35	31	31
Zinc	30	25	30	25	30	25	25
Copper	6	2.6	6	2.6	6	2.6	2.6
Selenium	0.15	0.2	0.15	0.2	0.15	0.2	0.2
*Vitamins (unit/kg)*							
Thiamin (mg)	5	3.5	5	3.5	5	3.5	3.5
Riboflavin (B_2_) (mg)	6	4.4	6	4.4	6	4.4	6
Niacin (mg)	30	50.6	30	50.6	30	50.6	50.6
Pyridoxine (B_6_) (mg)	6	3.9	6	3.9	6	3.9	6
Folate (mg)	2	1.3	2	1.3	2	1.3	2
Vitamin B_12_ (μg)	25	11	25	11	25	11	25
Vitamin A (IU)	4000	4300	4000	4300	4000	4300	4300
Vitamin D (IU)	1000	391	1000	391	1000	1000	391
Vitamin E (IU)	75	24.6	75	24.6	75	24.6	24.6
Vitamin K (μg)	750	189	750	189	750	189	189
Choline (mg)	1027	648	1027	648	1027	648	1027

*Abbreviations*: *AIN* American Institution of Nutrition, *TWD* total Western diet, *DIO* diet-induced obesity, *VMM* vitamin- and mineral-modified diet; *MM* macronutrient-modified diet; *TWD + CaVD* total Western diet with restored calcium and vitamin D; *TWD + MD* total Western diet with restored methyl donor micronutrients B_2_, B_6_, B_12_, choline and folate. *Notes*: Composition of the TWD was published previously [23]. No data are available in NHANES for chloride, manganese, iodine, pantothenic acid, biotin, or ultra-trace minerals. Thus, levels of these components in the AIN93G diet were used in the formulation of all experimental diets.

## Supplementary Materials

The following materials are available online at https://www.mdpi.com/2072-6643/12/2/544/s1, Figure S1: Design of animal model experiments; Figure S2: Impact of test diets on energy intake, body weight gain and colon length (Experiment A); Figure S3: Impact of test diets on energy intake, body weight, body composition, cecum weight and colon length (Experiment B); Figure S4: Effects of test diets, DSS treatment and sex on energy intake, body weight, body composition, colon length and cecum weight (Experiment C); Figure S5: Comparison of AIN and TWD diets on energy intake, body weight and body composition over time (Experiment D); Figure S6: Violin plots depicting distribution of normalized expression of the NanoString PanCancer Immune gene panel for Experiment E; Figure S7: Principal components and hierarchical clustering analyses of all genes identified as differentially expressed for any of pairwise comparisons made, including by diet at each time point, by time point for AIN diet, and by time point for TWD diet; Figure S8: Time course of significant differentially expressed genes associated with adaptive immune response, antigen processing, CD molecules and inflammation in colon tissues of mice prior to, during and after DSS-induced gut injury; Figure S9. Time course of significant differentially expressed genes associated with innate immune response, interferon, MHC, B-cell functions, and T-cell functions in colon tissues of mice prior to, during and after DSS-induced gut injury; Figure S10: Global and directed significance scores for immune-related pathways over the course of onset of colitis and recovery from gut injury; Figure S11: Circos plots and ontology terms for differentially expressed genes at colitis and recovery time points for AIN and TWD diets; Figure S12: Circos plots and ontology terms for differentially expressed genes unique to TWD at colitis and recovery time point; Figure S13: Network visualization of significant differentially expressed immune response genes at colitis as compared to pre-DSS time point in colon tissues of mice fed AIN; Figure S14: Network visualization of significant differentially expressed immune response genes at colitis as compared to pre-DSS time point in colon tissues of mice fed TWD; Figure S15: Network visualization of significant differentially expressed immune response genes at recovery as compared to pre-DSS time point in colon tissues of mice fed AIN; Figure S16: Network visualization of significant differentially expressed immune response genes at recovery as compared to pre-DSS time point in colon tissues of mice fed TWD; Figure S17: Changes in gene expression during colitis mapped to KEGG inflammatory bowel disease (mmu05321); Figure S18: Changes in gene expression at recovery mapped to KEGG inflammatory bowel disease (mmu05321); Figure S19: Changes in gene expression during colitis mapped to KEGG cytokine-cytokine receptor interaction pathway (mmu04060); Figure S20: Changes in gene expression during recovery mapped to the KEGG cytokine-cytokine receptor interaction pathway (mmu04060); Figure S21: Changes in gene expression during colitis mapped to the KEGG NF-kappa B signaling pathway (mmu04151); Figure S22: Changes in gene expression during recovery mapped to the KEGG NF-kappa B signaling pathway (mmu04151); Figure S22: Changes in gene expression during colitis mapped to the KEGG cell adhesion pathway (mmu04514); Figure S23: Changes in gene expression during recovery mapped to the KEGG cell adhesion pathway (mmu04514); Table S1: Comparison of colitis-responsive genes across animal models and to human ulcerative colitis. Supplemental data files are freely available at the Utah State University Digital Commons repository online at https://doi.org/10.26078/q4x4-6894, including File S1: Probe annotations.csv; File S2: Differential expression results.xlsx; File S3: Gene set analyses.xlsx; File S4: Pathway Z scores.xlsx; File S5: String-db networks and clusters.xlsx; File S6: Metascape ontology results.xlsx; File S7: Cell type scores.xlsx.
